# Multi-omics analysis reveals the physiological and molecular response to cold stress in different spring wheat cultivars at the booting stage

**DOI:** 10.3389/fpls.2025.1594676

**Published:** 2025-08-04

**Authors:** Miao Liu, Xiaoli Wu, Ming Li, Shizhao Li, Tao Xiong, Chaosu Li, Yonglu Tang

**Affiliations:** ^1^ Crop Research Institute of Sichuan Academy of Agricultural Sciences/Crop Germplasm Innovation and Genetic Improvement Key Laboratory of Sichuan Province/ Key Laboratory of Wheat Biology and Genetic Improvement on Southwestern China (Ministry of Agriculture and Rural Affairs), Chengdu, China; ^2^ Sichuan Provincial Key Laboratory of Water-Saving Agriculture in Hill Areas of Southern China, Chengdu, China; ^3^ Key Laboratory of Crop Ecophysiology and Farming System in Southwest China, Ministry of Agriculture, Chengdu, China

**Keywords:** wheat, cold stress, physiological indicators, transcriptome, proteome, metabolome

## Abstract

**Introduction:**

Cold stress at the booting stage can seriously affect wheat growth, development and yield.

**Methods:**

Therefore, this study employed integrated physiological, transcriptomic, proteomic and metabolomic approaches to examine the response of two wheat cultivars, Chuanmai 104 (CM104, cold-tolerant) and Chuanmai 42 (CM42, cold-sensitive), to cold stress at the booting stage.

**Results:**

The viability of pollen in CM104 was less affected by low-temperature stress compared to CM42, ensuring a higher seed-setting rate in CM104. The young spike of CM104 also synthesized more osmoregulatory substances, endogenous hormones and higher antioxidant enzyme activities under the cold treatment compared to CM42. Transcriptome analysis identified 7,362 and 5,328 differentially expressed genes (DEGs) between control and cold-treated CM104 and CM42 spike samples, respectively. More DEGs, such as transcription factors, late embryogenesis abundant protein and hormone signalling transduction involved in the key regulatory pathways associated with cold tolerance were expressed in CM104. Proteomic and metabolomic analyses identified 173 differentially expressed proteins and 180 differentially accumulated metabolites between control and cold-treated CM104 spike samples, with some thought to enhance the cold acclimation of the variety. Integrative multi-omics analysis highlighted the critical roles of starch and sucrose, and glycerophospholipid metabolism in response to cold stress in CM104.

**Discussion:**

This study uncovered the physiological changes, gene, protein and metabolite pathways involved in maintaining the osmotic balance and mitigating low-temperature stress in wheat spikes, and could serve as a crucial reference for selecting and breeding low-temperature tolerant wheat varieties.

## 1 Introduction

Global food security is severely affected by the recent changes in climate due to many extreme weather events (FAO, 2022). Over the past few decades, the major agricultural regions of the world have experienced more extreme cold or heat of varying frequency, intensity and duration (Liu et al., 2023a). Cold stress induces physiological and biochemical changes in crops, negatively impacting their growth and development (Hassan et al., 2021). Wheat (*Triticum aestivum* L.) is one of the important cereal crops, that provide a staple food for over half of the world population (FAO, 2022). However, cold stress usually hampers its normal growth and yield through mechanical damage and disruption of crystal metabolic activity (Yadav, 2010). Moreover, global warming accelerates the early growth of wheat and heightens the risk of spring cold injury (Li et al., 2016). For instance, in the main wheat-growing regions of China, including the Huang-Huai and Yangtze-Huai River Basins, low temperatures in spring often occur between March and April during its booting stage, in which its spikes undergo meiosis and form tetrads, making it hypersensitive to temperature stress (Thakura et al., 2010). The cold stress also disrupts its pollen and ovule development, decreasing its seed-setting rates and potentially reducing final grain production by 30-50% under severe conditions (Thakura et al., 2010; Ji et al., 2017).

Therefore, more adaptation strategies for agriculture are necessary in the face of the current complex climate extremes. These strategies include proper management and institutional food-system interventions, soil characterisation and breeding of new crop varieties and genetics (Liu et al., 2023a). Among these, breeding and promotion of cold-tolerant wheat cultivars are the most economical and effective way to keep yields stable and reduce the negative effects of sudden extreme cold events (Limin and Fowler, 2006). However, this requires the need to explore cold-resistant germplasm resources and carry out an in-depth analysis of the mechanisms involved in resisting low temperatures (Frascaroli, 2018). These mechanisms are very complex and may include physiological, biochemical and molecular responses of related gene expression and regulation networks (Yadav, 2010; Hassan et al., 2021). For example, under cold stress, wheat shows changes in membrane fluidity, reduced tissue water content, improved antioxidant enzyme activity, and increased contents of soluble substances and phytohormones (Zhang et al., 2016; Hail et al., 2017).

Besides, omics technology such as transcriptomics, proteomics and metabolomics offers a comprehensive perspective for elucidating molecular and biological issues in crops under diverse environmental stresses. For instance, transcription factor (TF) families, and other genes involved in starch and sucrose, and glutamate metabolism, and phytohormone signalling pathways contribute to the different cold tolerance capacities in wheat cultivars (Jiang et al., 2022; Ahad et al., 2023; Li et al., 2023). Cold tolerance was also enhanced in the wheat crowns by regulating proteins associated with carbohydrate metabolism, stress and defense response, signal transduction and phenylpropanoid biosynthesis (Xu et al., 2022). The combined transcriptome and metabolome analysis also identified abscisic acid (ABA)/jasmonic acid (JA), signal transduction, sucrose and amino acid, and flavonol biosynthesis as key pathways in regulating cold resistance in wheat (Zhao et al., 2019; Lv et al., 2022).

Despite this significant progress, to date, there are few comprehensive studies on the physiological changes, gene and protein expression levels and metabolite accumulation related to the mechanisms that regulate cold tolerance in wheat at the booting stage. Therefore, we examined the morphological and physiological changes in cold-tolerant and cold-sensitive wheat cultivars under varying durations of cold stress and identified key genes, proteins and metabolites involved in response to cold stress using transcriptomics, proteomics and metabolomics. We also determined the key regulatory networks associated with cold tolerance to characterize the molecular responses involved in the adaptation of wheat spike to cold stress. These results could enrich and improve the physiological and molecular theory of wheat stress tolerance, provide valuable genetic information for the study of wheat cold resistance mechanisms, and lay a foundation for breeding new wheat varieties with high cold resistance.

## 2 Materials and methods

### 2.1 Plant materials and cold treatments

The study was conducted between 2022 and 2023 at the experimental base of the Sichuan Academy of Agricultural Sciences (104°39′E, 31°00′N) located in Guanghan City, Sichuan Province, China. Two wheat cultivars, Chuanmai 104 (CM104, cold-tolerant) and Chuanmai 42 (CM42, cold-sensitive), which exhibit similar growth but differ in cold tolerance at the booting stage were sown in 24 x 21 cm pots on October 30, 2022 and harvested on May 5, 2023. The seeds of both cultivars used in the experiments were kindly provided by the Crop Research Institute of Sichuan Academy of Agricultural Sciences, China. CM42 was bred by using first-generation synthetic hexaploid wheat (Syn 769, Decoy 1/Aegilops tauschii 188) and CM104 was derived from CM42 (Liu et al., 2021). The soil used was composed of pH 7.46, organic matter of 31.76 g kg^-1^, total nitrogen (N) of 1.98 g kg^-1^, available N of 118.80 mg kg^-1^, total phosphorus (P) of 1.64 g kg^-1^, available P of 68.05 mg kg^-1^, total potassium (K) of 16.68 g kg^-1^ and available K of 157.75 mg kg^-1^. Exactly 7 kg of soil was mixed with 4.1 g of NPK compound fertilizer in a ratio of 15:15:15 in each pot and used to sow 24 seeds per pot, with 16 seedlings retained post-emergence. The seedlings were then placed in the natural field until the plants reached the booting stage. The relative water content of the soil was maintained at 60-70% throughout the whole growth period. At the booting stage, the stems that grew uniformly were marked. All pots, except for the control treatments were then transferred to a smart climate chamber (Nanjing Hengyu Instruments and Equipments Co., Ltd, Nanjing, China) with 70% relative humidity and white light conditions (500 μmol m^−2^ s^−1^ during the day and 0 μmol m^−2^ s^−1^ at night). There were 100 pots for each cultivar, with one control (CK) and three cold stress treatments. The first treatment (T1) consisted of plants kept at 4 °C for 16h in light (L) and at -1 °C in darkness (D) once, while the second (T2) and third treatments (T3) consisted of plants maintained at the same conditions for 3 and 5 days, respectively. Spike tissue samples from 24 individual stems per treatment were pooled in four replicates after the cold treatment, immediately frozen in liquid nitrogen, and stored at −80°C for subsequent physiological and molecular analyses. Of which, molecular analyses (transcriptome, proteome and metabolome) were only carried out in the T3 treatment due to the more obvious phenotypic differences between the two varieties under this treatment. The remaining plants were transferred to the natural field environment to assess pollen viability at anthesis and seed setting rate at the maturity stage. Meteorological data (air temperatures and precipitation) during the field trial period were collected from a meteorological station adjacent to the field experiment site (**Supplementary Figure 1**).

### 2.2 Determination of pollen viability and seed setting rate

Pollen viability at anthesis was assessed using the 2,3,5-triphenyl tetrazolium chloride (TTC) staining method (Fábián et al., 2019). Fresh pollen was collected from the base and top of the spikes of different treatments and incubated in a 1.5 ml centrifuge tube with 0.1% TTC for 30 minutes at 37°C. The stained pollen was examined for viable and non-viable pollen grains under an Olympus BX51 light microscope (Olympus, Tokyo, Japan) at room temperature. The pollen abortion rate (%) was calculated by dividing the number of colorless pollen grains by the total pollen grains counted x 100%. A minimum of 1,000 pollen grains were studied for different varieties and treatments in triplicates.

At the maturity stage, 60 marked stems were harvested in triplicates for each genotype and treatment and used to record the fertility of superior florets per spikelet, the number of degenerate and fertile spikelets per spike, and the thousand-grain weight. The superior florets were defined as the two florets proximal to the rachis per spikelet, which usually carry seeds (Zheng et al., 2016). The seed setting rate of superior florets per spike (%) was calculated by dividing the seed number of superior florets per spike by two times the number of fertile spikelets per spike x 100%.

### 2.3 Determination of physiological indicators

The contents of soluble sugar and protein, proline, ABA and gibberellin (GA_3_), and the activities of catalase (CAT, EC 1.11.1.6), superoxide dismutase (SOD, EC 1.15.1.1), peroxidase (POD, EC 1.11.1.7) in young spikes under different cold treatments were measured using different assay kits from Suzhou Comin Biotechnology Co. Ltd. (Suzhou, China).

### 2.4 Transcriptome sequencing and analysis

Total RNA was extracted from the young spike tissues of the control and T3 cold-treated CM104 and CM42 genotypes in triplicates using the RNeasy Plant Mini Kit (Qiagen Inc, Germany) and their quantities and qualities were evaluated using NanoDrop 2000 (Thermo Fisher Scientific, USA). Sequencing libraries were generated using the NEBNext Ultra™ RNA Library Prep Kit for Illumina (NEB, USA) and sequenced on an Illumina HiSeq 2500 platform (Beijing Biomarker Technologies Co., LTD., China). The in-house Perl scripts were used to remove the adapter, ploy-N and low-quality reads. These clean reads were then aligned to the wheat reference genome IWGSC RefSeq v2.1 (https://urgi.versailles.inra.fr/download/iwgsc/IWGSC_RefSeq_Assemblies/v2.1/) using HISAT2 v2.2.1 (http://daehwankimlab.github.io/hisat2/). Bowtie2 v2.5.0 (https://bowtie-bio.sourceforge.net/bowtie2/) and StringTie v2.2.0 (https://ccb.jhu.edu/software/stringtie/) were used to align the clean reads and calculate gene expression profiles, respectively. Gene function was annotated based on Nr (NCBI non-redundant protein sequences), Nt (NCBI non-redundant nucleotide sequences), KO (KEGG Ortholog database), GO (Gene Ontology), Pfam (Protein family), Swiss-Prot (A manually annotated and reviewed protein sequence database), and KOG/COG (Clusters of Orthologous Groups of proteins). Gene expression levels were quantified in FPKM (fragments per kilobase of transcript per million mapped fragments). Differentially expressed genes (DEGs) analysis was conducted using the DESeq2 v1.26.0 R package (https://github.com/thelovelab/DESeq2). The DEGs were identified using the threshold of |log2FoldChange| ≥ 1 and false discovery rate (FDR) < 0.01. Enrichment analysis of the DEGs for GO and KEGG pathways was conducted using GOseq v1.26.0 R package (https://github.com/lmika/goseq) and KOBAS v2.0 (http://kobas.cbi.pku.edu.cn/), respectively.

### 2.5 Quantitative real-time polymerase chain reaction validation

To validate the transcriptome data, 16 DEGs associated with cold stress were randomly selected for verification by quantitative real-time polymerase chain reaction (qRT-PCR) analysis in CM104 and CM42. The detailed primer sequences used are listed in **Supplementary Table 1**. The wheat actin gene was used as an internal reference gene. The qRT-PCR was conducted on an ABI7500 Real-Time PCR System (Applied Biosystems, USA) with a total of 20 μL volume, containing 10 μL SYBR Green PCR-mix (DF Biotech., China), 2.5 μL of cDNA, 5.5 μL ddH_2_O, and 1 μL of each primer. The PCR reactions were conducted at 95°C for 3 min, followed by 40 cycles of 95°C for 10 s, 60°C for 30 s, and 72°C for 30 s. The relative gene expression levels of the DEGs were determined by the 2^^-△△Ct^ method (Livak and Schmittgen, 2001) in triplicates.

### 2.6 Proteome analysis

To further investigate the protein expression of cold-tolerant variety under cold stress, protein was extracted from young spikes of the CK and T3 cold-treated CM104 wheat in triplicates using the TCA-acetone precipitation method (Jorrin-Novo, 2014). Total protein concentration was measured by the Bradford Protein Assay Kit and the proteome was analysed using the tandem mass tags technology from Biomarker Tech. Co. (Beijing, China), as described by McLoughlin et al. (2018). The MS/MS raw data were analyzed using the MASCOT engine v2.2 (Matrix Science, UK) which is integrated into Proteome Discoverer 1.4 (Thermo Fisher Scientific, USA). Searches utilized a peptide mass tolerance of 15 ppm and a production tolerance of 0.02 Da, achieving a 5% FDR. Proteins were annotated using the KEGG and COG databases for functional annotation and enrichment analyses, respectively. Differentially expressed proteins (DEPs) were identified using the Student’s t-test (P < 0.05) and a fold-change threshold of >1.5 or <0.67.

### 2.7 Metabolome analysis

To further evaluate the metabolite accumulation of cold-tolerant variety under cold stress, metabolites were extracted from spike samples of CK and T3 cold-treated CM104 in triplicates and processed for metabolomics analysis using the LC-MS system as described previously (Liu et al., 2023b). Raw data was collected using MassLynx V4.2. The data processing operations were processed by the Progenesis QI software based on the METLIN database and Biomark’s self-built library for identification. The classification and pathway information of identified compounds were searched in KEGG, HMDB and lipidmaps databases. The significant difference in each compound was determined by Student’s t-test. Orthogonal partial least squares discriminant analysis (OPLS-DA) modelled by the R language package “ropls” was used. The variable importance in the projection (VIP) value of the model was determined through multiple cross-validation. Differentially accumulated metabolites (DAMs) were identified by combining the different multiple, p-values, and the VIP value of the OPLS-DA model, with the following criteria: fold-change ≥ 1, p< 0.05, and VIP > 1. Finally, the significance of the DAMs in the KEGG pathway enrichment was assessed using a hypergeometric distribution test.

### 2.8 Statistical analysis

Agronomic and physiological data of the two cultivars under different cold treatments were analyzed by one-way analysis of variance with SAS, version 9.4 (SAS Institute, Cary, NC). Duncan’s multiple range test was performed to determine the statistically significant differences. Comparison of treatment/variety means was based on least significant difference (LSD) at a 0.05 probability level. Plotting was performed using Origin 2021 and Microsoft Office Excel 2020.

## 3 Results

### 3.1 Effect of cold stress on the agronomic and physiological traits of different wheat varieties

**Figure 1:**
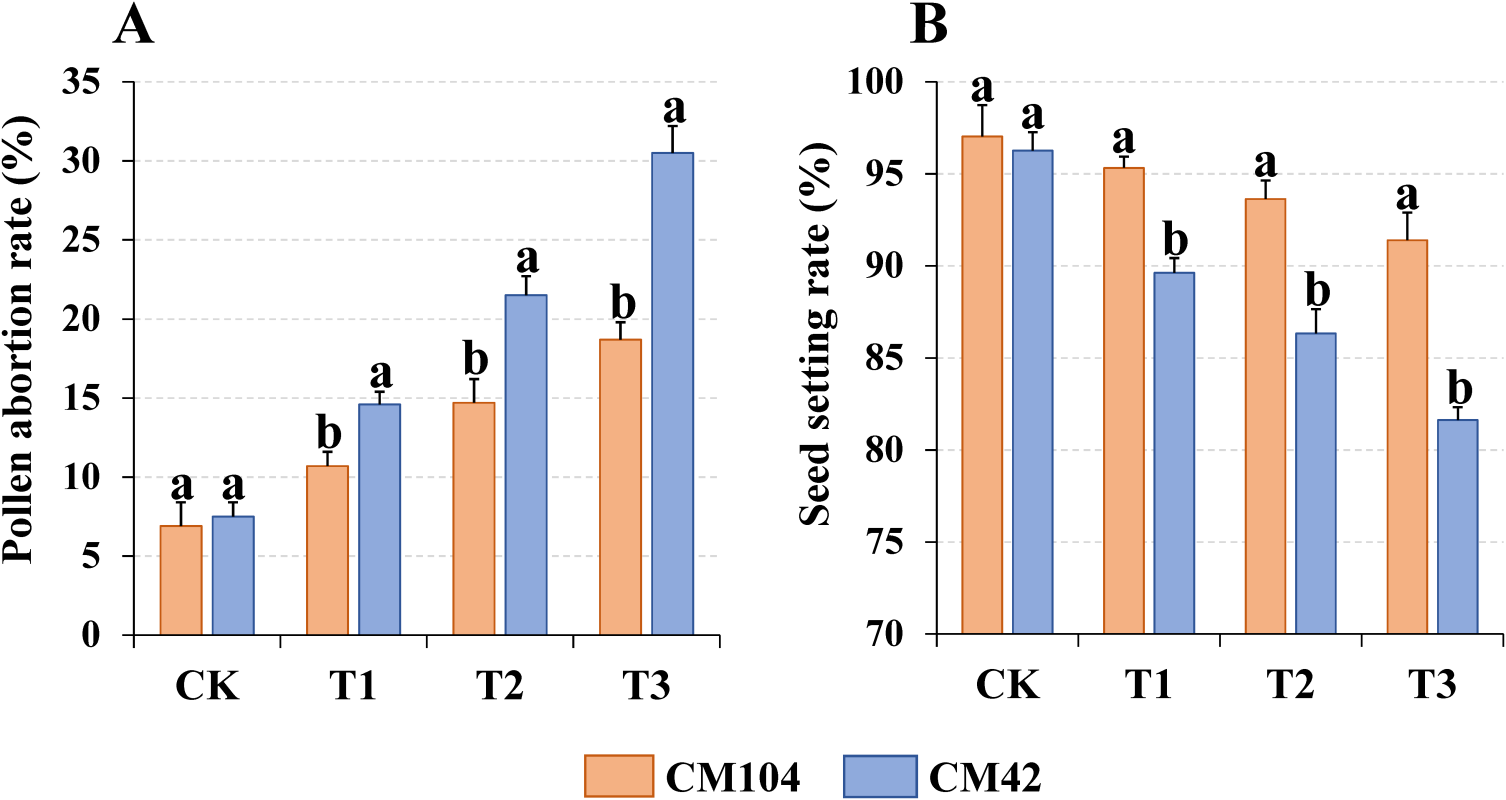
Pollen abortion rate and seed setting rate of Chuanmai 104 (CM104) and Chuanmai 42 (CM42) under different cold treatments. **(A)** Pollen abortion rate, **(B)** Seed setting rate. CK, control; T1, 1 day; T2, 3 days, T3, 5 days. Vertical bars are standard errors. Different lowercase letters on the columns indicate significant differences between CM104 and CM42 under the same treatment based on one-way analysis of variance with Duncan’s multiple range test (*P* < 0.05).

Different cold stresses had a significant effect on the pollen viability and seed-setting rate of the two wheat varieties. The pollen abortion rates at the anthesis significantly increased in all three treatments (T1, T2 and T3) in both wheat varieties compared to the controls, with more abortion rates in T3-treated samples (**Figure 1A**; **Supplementary Figure 2**) and higher pollen abortion rates in CM42-treated samples than in CM104. The cold stress significantly reduced the seed-setting rates in both varieties at the maturity stage, with lower rates in T1-, T2- and T3-treated CM42 samples than in CM104 cold-treated samples (**Figure 1B**; **Supplementary Figure 3**). However, there were no significant differences in the number of degenerate and fertile spikelets per spike, or thousand-grain weights across different cold treatments for each variety (**Supplementary Table 2**).

**Figure 2:**
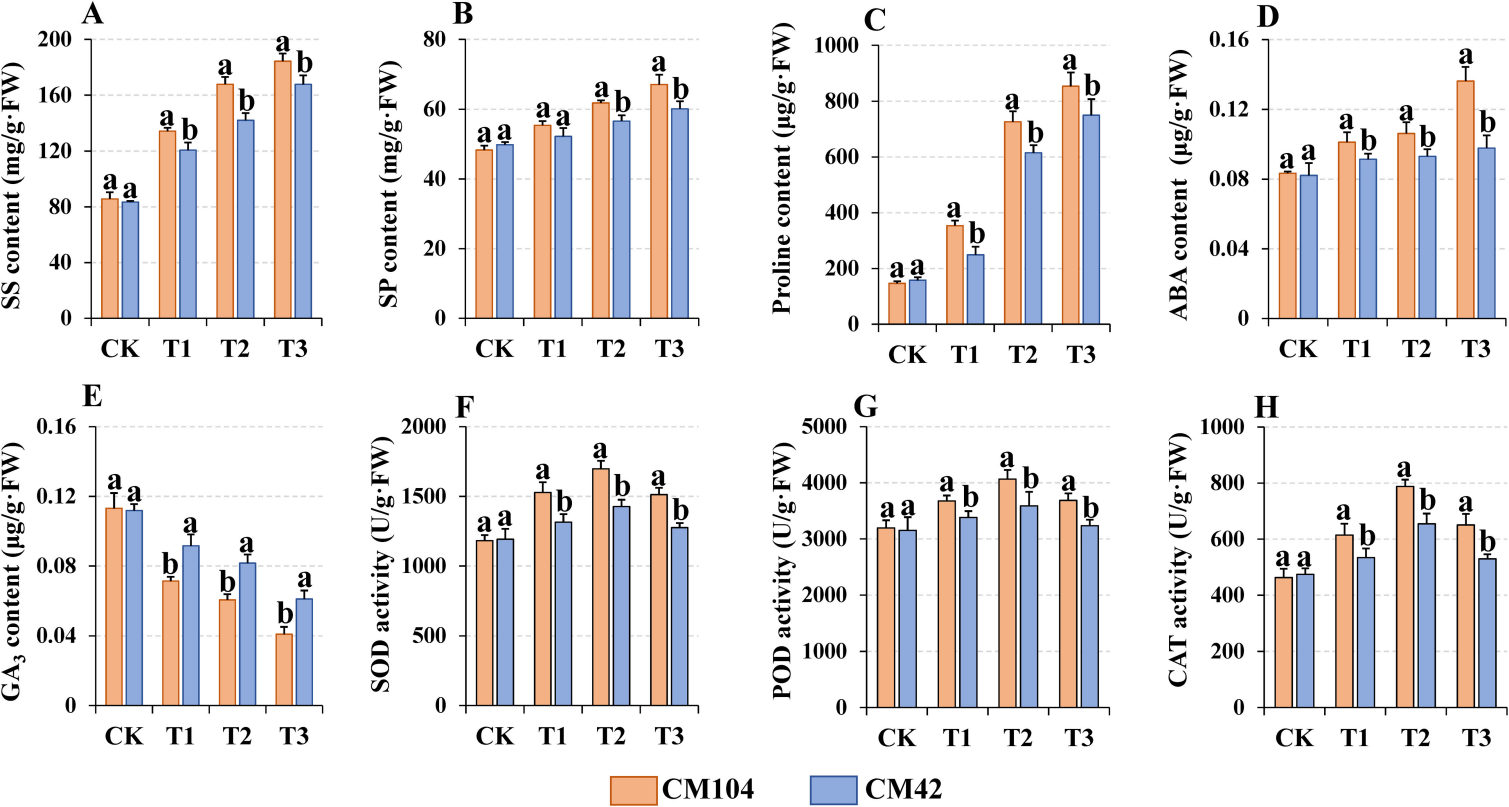
Physiological indicators of Chuanmai 104 (CM104) and Chuanmai 42 (CM42) under different cold treatments. CK, control, T1, 1 day, T2, 3 days, T3, 5 days. **(A)** Soluble sugar content, **(B)** Soluble protein content, **(C)** Proline content, **(D)** Abscisic acid (ABA) content, **(E)** Gibberellin (GA_3_) content, **(F)** Superoxide dismutase (SOD) activity, **(G)** Peroxidase (POD) activity, and **(H)** Catalase (CAT) activity. Vertical bars are standard errors. Different lowercase letters on the columns indicate significant differences between CM104 and CM42 under the same treatment based on one-way analysis of variance with Duncan’s multiple range test (*P* < 0.05).

The contents of the soluble and sugar protein, proline and ABA showed a significant upward trend from T3-, T2-, and T1-treated samples in both varieties to the controls, with those detected in CM104 also significantly higher than those found in CM42 (**Figures 2A–D**). On the contrary, GA_3_ content significantly declined in both varieties, with CM104 showing a markedly lower hormone than CM42 under the same cold treatment (**Figure 2E**). The antioxidant enzyme activities of SOD, POD, and CAT initially increased and then decreased with prolonged cold treatment, with CM104 exhibiting higher enzyme activities than CM42 under the same cold treatment (**Figures 2F–H**).

### 3.2 Transcriptome analysis of genes involved in response to cold tolerance in wheat genotypes under cold stress

#### 3.2.1 Illumina sequencing data

From the 12 libraries, 81.31 GB of clean data and approximately 272 million reads were obtained, averaging 6.78 GB and 23 million reads per sample. The GC content varied between 53.32 and 54.79%, with an average Q30 percentage exceeding 94.05% and the percentage of unique mapped reads exceeding 89.47% (**Supplementary Table 3**). The mapped read results showed 15,267 new genes, with 3,897 having functional annotations (**Supplementary Table 4**). A strong correlation was also evident among all three biological replicates of the different samples, as indicated by the Pearson correlation coefficients (*R* ^2^ = 0.88-0.98) and principal component analysis map (**Supplementary Figure 4**). These results indicated that the sequencing data sufficiently met the quality requirements for further analysis. The raw transcriptome data are accessible in the NCBI database (Accession number: PRJNA1131989).

#### 3.2.2 Functional analysis of differently expressed genes

**Figure 3:**
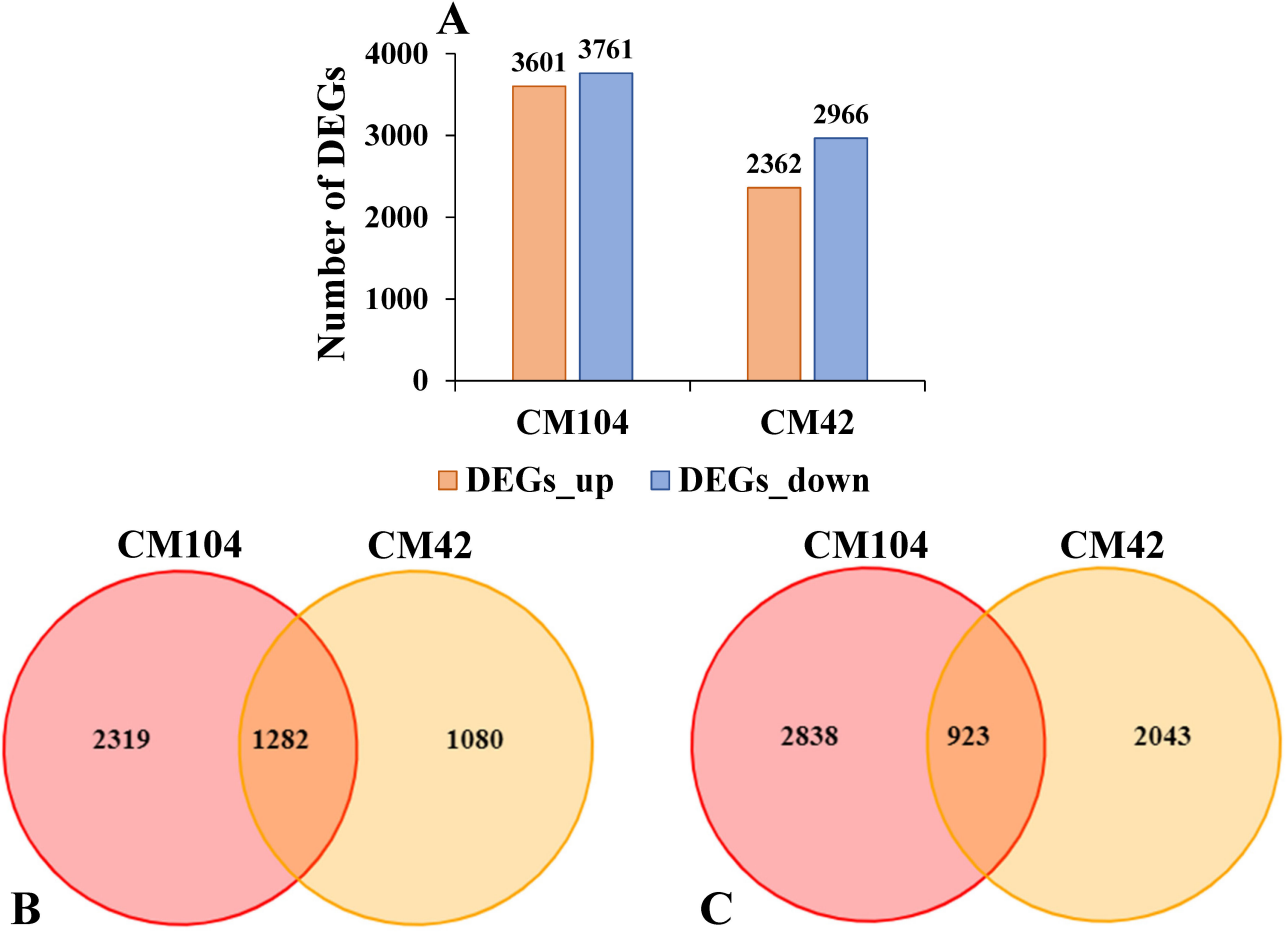
Overview of the transcriptome analysis. **(A)** Number of differentially expressed genes (DEGs). **(B)** Venn diagram for the up-regulated DEGs after the cold treatment of Chuanmai 104 (CM104) and Chuanmai 42 (CM42). **(C)** Venn diagram for the down-regulated DEGs after the cold treatment of CM104 and CM42. CM104CK, CM104 at the control treatment, CM104CT, CM104 after the cold treatment, CM42CK, CM42 at the control treatment, CM42CT, CM42 after the cold treatment.

Gene expression profiles of CM104 and CM42 were analyzed using FPKM values to identify key genes related to cold stress. A total of 7,362, including 3,601 up-regulated and 3,761 down-regulated, and 5,328 consisting of 2,362 up-regulated and 2,966 down-regulated DEGs were identified between the CK vs cold-treated CM104 (CM104CK_vs_CM104CT) and CK vs. CM42 (CM42CK_vs_CM42CT) spike samples, respectively (**Figure 3A**). Among the up-regulated genes, 1,282 were common to both groups, while 2,319 and 1,080 genes were uniquely identified in CM104CK_vs_CM104CT and CM42CK_vs_CM42CT, respectively. Among the down-regulated, 923 genes were common to both groups, while 2,838 and 2,043 genes were uniquely identified in CM104CK_vs_CM104CT and CM42CK_vs_CM42CT, respectively (**Figures 3B, C**).

**Figure 4:**
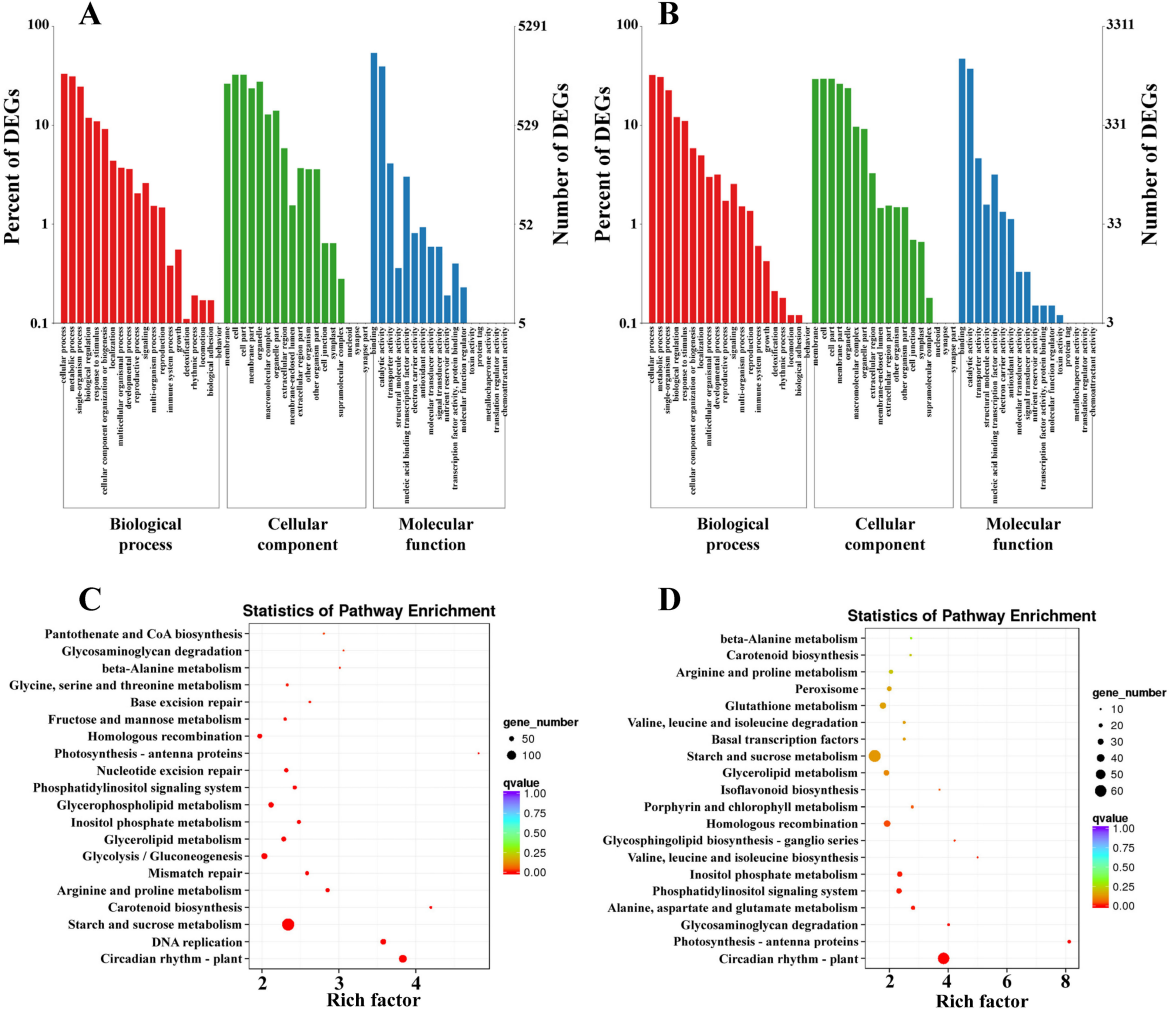
GO and KEGG pathway enrichment analysis for differentially expressed genes (DEGs) in two comparison groups. **(A)** GO analysis for Chuanmai 104 (CM104), **(B)** GO analysis for Chuanmai 42 (CM42), **(C)** KEGG pathway enrichment analysis for CM104, and **(D)** KEGG pathway enrichment analysis for CM42.

To examine the functions of DEGs involved in response to low temperatures between CM104/CM42 and the controls, the DEGs were annotated in COG, GO, KEGG and KOG (**Supplementary Table 5**). Based on the GO annotations, 5,291 and 3,311 DEGs between CM104CK_vs_CM104CT and CM42CK_vs_CM42CT were classified into 49 and 51 functional subgroups, including 19 and 19 in the biological process, 16 and 18 in molecular function, 14 and 14 in cellular component, respectively (**Figures 4A, B**). The primary categories of biological process predominantly associated DEGs with cellular, metabolic, and single-organism processes, while for cellular component, the majority of categories were associated with membranes, cells, and cell parts. For molecular function, the DEGs were associated with binding, catalytic and transporter activities (**Supplementary Table 6**). KEGG analysis revealed that DEGs were significantly associated with 130 and 125 metabolic pathways between CM104CK_vs_CM104CT and CM42CK_vs_CM42CT, respectively (**Supplementary Table 7**). In CM104CK_vs_CM104CT, the top three enriched pathways were; plant circadian rhythm, DNA replication, and starch and sucrose metabolism (**Figure 4C**). In CM42CK_vs_CM42CT, the top three enriched pathways were; plant circadian rhythm, photosynthesis - antenna proteins, and glycosaminoglycan degradation (**Figure 4D**).

#### 3.2.3 Identification of key DEGs associated with cold response

The TFs are crucial regulators of plant resistance to abiotic stresses. In this study, we identified 203 and 114 TFs in CM104CK_vs_CM104CT and CM42CK_vs_CM42CT, respectively, with 54 common in both cultivars (**Supplementary Table 8**). The bHLH, AP2/ERF and heat shock factors (HSFs) were the three largest TF families, while three identical DEGs associated with NAC TFs were up-regulated in both cultivars. Two CSA TFs and two MTB2 TFs were uniquely identified in CM104, while the DP and LG2 TFs were uniquely identified and up-regulated in CM42.

Late embryogenesis abundant (LEA) protein is a class of small protective hydrophilic proteins that protect macromolecules and cellular structures to enhance plant tolerance to abiotic stress (Soualiou et al., 2022). Dehydrin, a unique LEA protein group, plays a role in plant response to low temperatures. This study identified 92 and 36 significant LEA-related DEGs in CM104CK_vs_CM104CT and CM42CK_vs_CM42CT, respectively, with 33 DEGs common to both cultivars (**Supplementary Table 9**). All the 24 DEGs associated with dehydrin in CM104, such as TraesCS7A03G1365300, TraesCS6A03G0899900 and TraesCS6A03G0899800 (log_2_FC > 10) were highly up-regulated, while 3 of 10 DEGs were down-regulated in CM42.

Phytohormones are crucial in plant signal transduction under abiotic stress. In the current study, 133 and 72 DEGs related to hormone signal transduction were identified in CM104CK_vs_CM104CT and CM42CK_vs_CM42CT, respectively, with 41 DEGs found in both cultivars (**Supplementary Table 10**). The majority of DEGs were found in the ABA, Auxin, and Ethylene signalling pathways. Some DEGs that are specific and might be involved in cold tolerance in CM104, are protein phosphatase 2C (PP2C), auxin transporter-like protein and ethylene-responsive factor genes. All brassinosteroid-related genes were down-regulated under cold stress, while four DEGs related to GA_3_ were uniquely up-regulated in CM104, and one DEG was down-regulated in CM42. The distinct expression of DEGs related to cold response in the two varieties likely plays a key role in cold stress adaptation, with the uniquely identified DEGs in CM104 being linked to its cold tolerance.

#### 3.2.4 Validation of differently expressed genes by qRT-PCR

The expression levels of 16 DEGs involved in calcium, carbohydrate transport, phytohormones, and transcription factors were measured using qRT-PCR, with the results mirroring the RNA-seq expression patterns (**Supplementary Figure 5**), confirming the accuracy and reliability of the RNA-seq data.

### 3.3 Proteome analysis of cold-tolerant wheat genotype CM104 under cold stress

**Figure 5:**
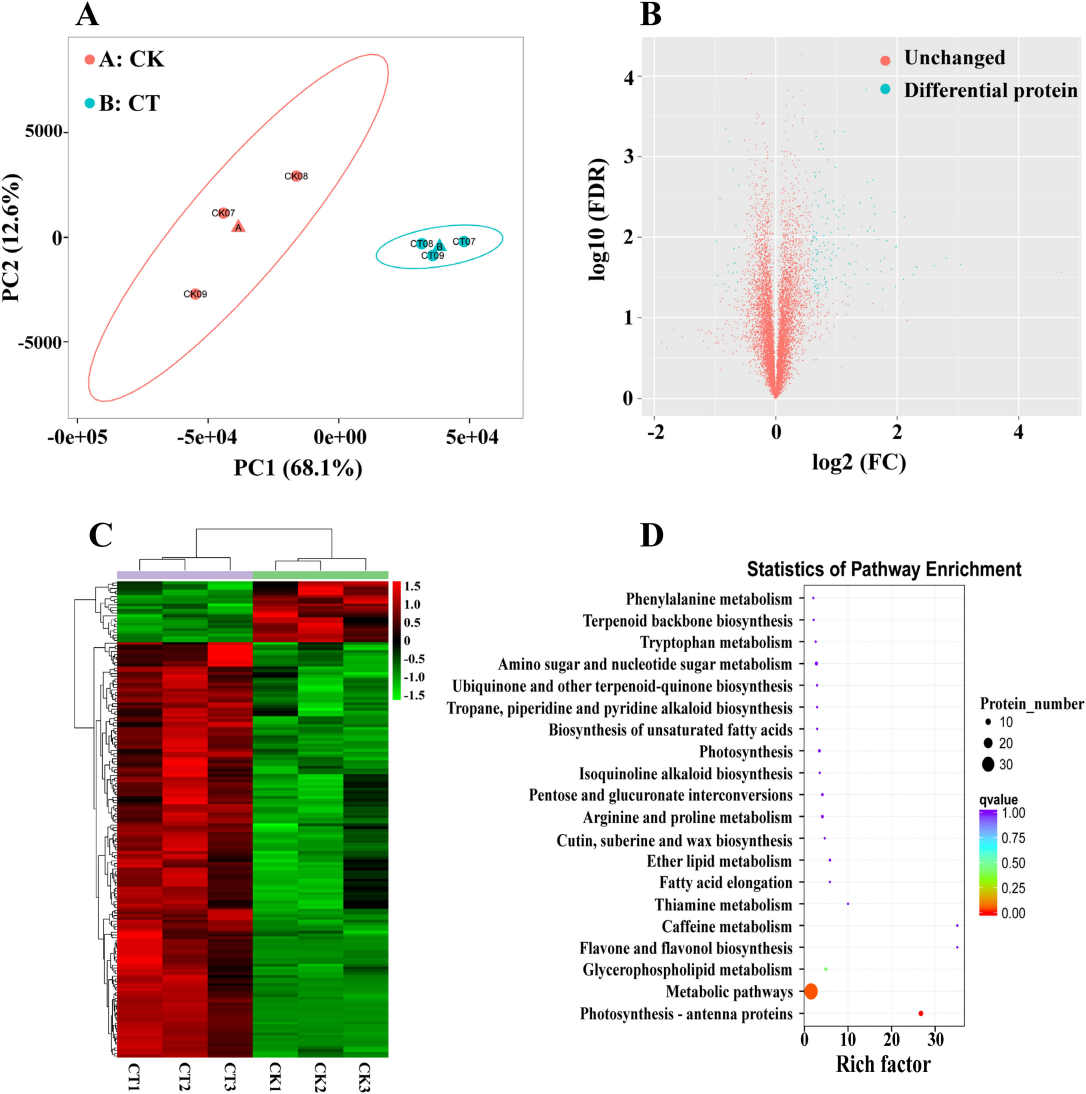
Proteomic analysis of Chuanmai 104 under cold stress. **(A)** Principal component analysis of all samples, **(B)** Volcano plot of differentially expressed proteins (DEPs), **(C)** Clustering heatmap of DEPs, and **(D)** KEGG pathway enrichment analysis of DEPs.

A total of 9,656 proteins were found and analyzed in CM104CK_vs_CM104CT (**Supplementary Table 11**). The principal component analysis of the proteome revealed a clear distinction between the two treatments (**Figure 5A**). The cold stress resulted in significant changes in 173 DEPs compared to CK, with 151 proteins upregulated and 22 proteins downregulated (**Figure 5B**). Of these, two dehydrin proteins, namely TraesCS7A03G1365300 and TraesCS7B03G1305200 were the most up-regulated DEPs (log_2_FC: 4.70 and 3.04). The abundance of several cold-responsive proteins, including TraesCS2D03G0959100 (log_2_FC: 2.80), TraesCS2B03G1138000 (log_2_FC: 2.30), and two LEA proteins, namely TraesCS5A03G1181100 (log_2_FC: 2.10) and TraesCS3A03G0416800 (log_2_FC: 2.00) also substantially increased (**Supplementary Table 12**). The results of hierarchical clustering algorithm analysis showed that the criteria for the identification of DEPs were reasonable (**Figure 5C**). The COG enrichment analysis revealed the participation of numerous DEPs in post-translational modification, protein turnover, chaperones, general function prediction, cell wall/membrane/envelope biogenesis, amino acid transport and metabolism, and lipid transport and metabolism (**Supplementary Figure 6**). The KEGG enrichment analysis also indicated significant changes in key pathways, including photosynthesis - antenna proteins, metabolic pathways and glycerophospholipid metabolism that were significantly changed (**Figure 5D**).

### 3.4 Metabolome analysis of cold-tolerant wheat genotype CM104 under cold stress

**Figure 6:**
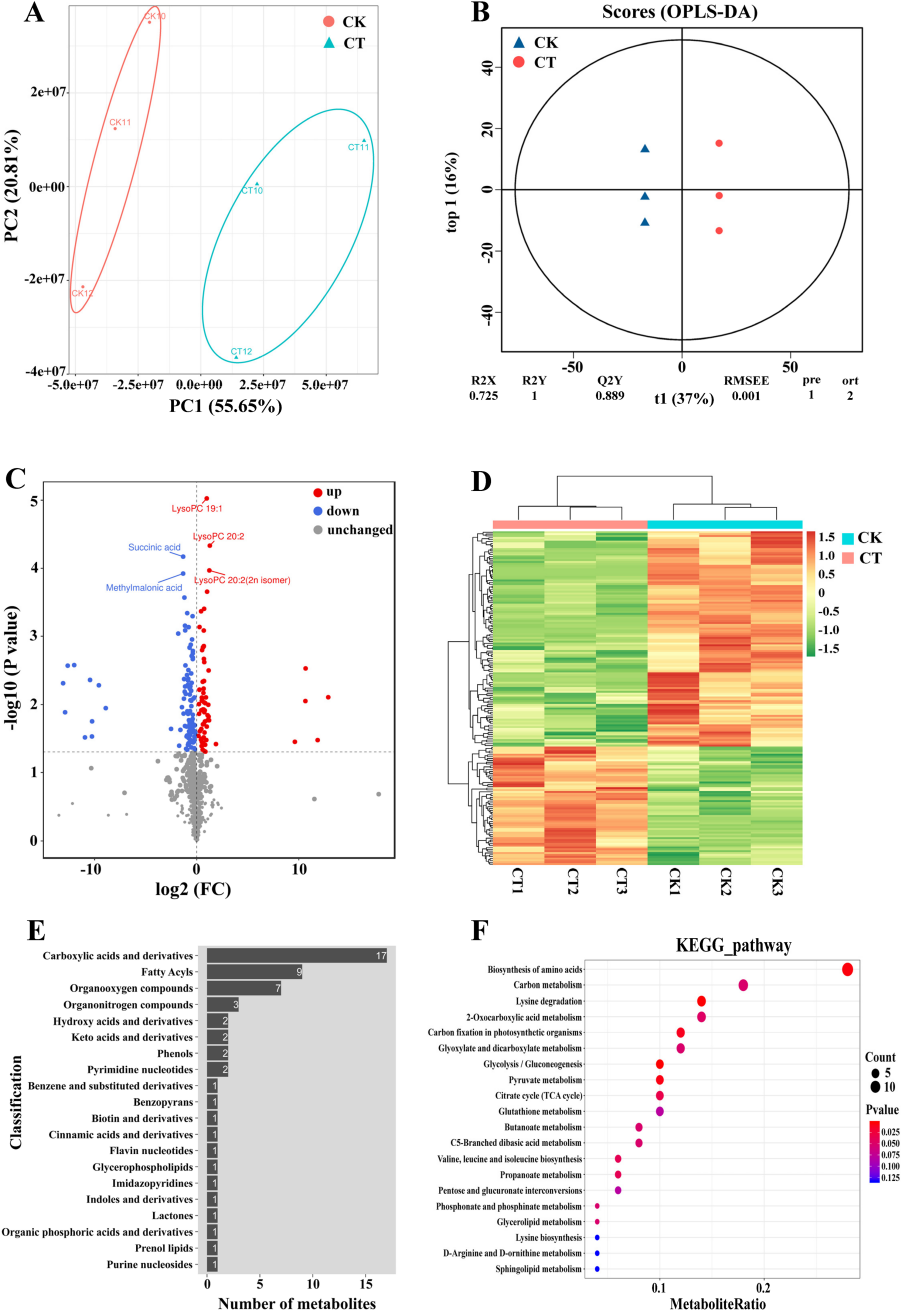
Metabolomic analysis of Chuanmai 104 under cold stress. **(A)** Principal component analysis of all samples, **(B)** OPLS-DA analysis of all samples, **(C)** Volcano plot of differentially accumulated metabolites (DAMs), **(D)** Clustering heatmap of DAMs, **(E)** HMDB classification of DAMs in each group, and **(F)** KEGG pathway enrichment analysis of DEPs.

A total of 938 metabolites were detected between CM104CK and CM104CT, with a decrease in the content of 586 and an increase in the content of 352 metabolites (**Supplementary Table 12**). The principal component analysis revealed a notable distinction in metabolic patterns between the CK and CT groups (**Figure 6A**). The OPLS-DA analysis indicated significant changes in metabolite concentrations under cold stress, suggesting that certain variables could be used to differentiate between the CK and CT groups (**Figure 6B**). A comparison of the metabolite concentrations between CM104CK and CM104CT groups identified 180 DAMs, with 64 upregulated and 116 downregulated (**Figures 6C, D**; **Supplementary Table 12**). The four most up-regulated DAMs were LysoPC 22:6, 6’-O-Sinapoylsucrose, 4-O-(2’’-O-acetyl-6’’-P-coumaroyl-β-D-glucopyranosyl)-P- coumaric acid and 5-L-Glutamyl-L-amino acid (log_2_FC>10, **Supplementary Table 12**). The DAMs were categorized into 21 groups, with carboxylic acids and their derivatives, fatty acyls, and organooxygen compounds as the three most abundant groups (**Figure 6E**). Additionally, KEGG enrichment analysis identified significant enrichment in 69 metabolic pathways, including amino acid biosynthesis, carbon metabolism, lysine degradation and 2-oxocarboxylic acid metabolism as the four most enriched pathways (**Figure 6F**).

### 3.5 Integrated analysis of multi-omics data responding to cold stress in cold-tolerant wheat genotype CM104

**Figure 7:**
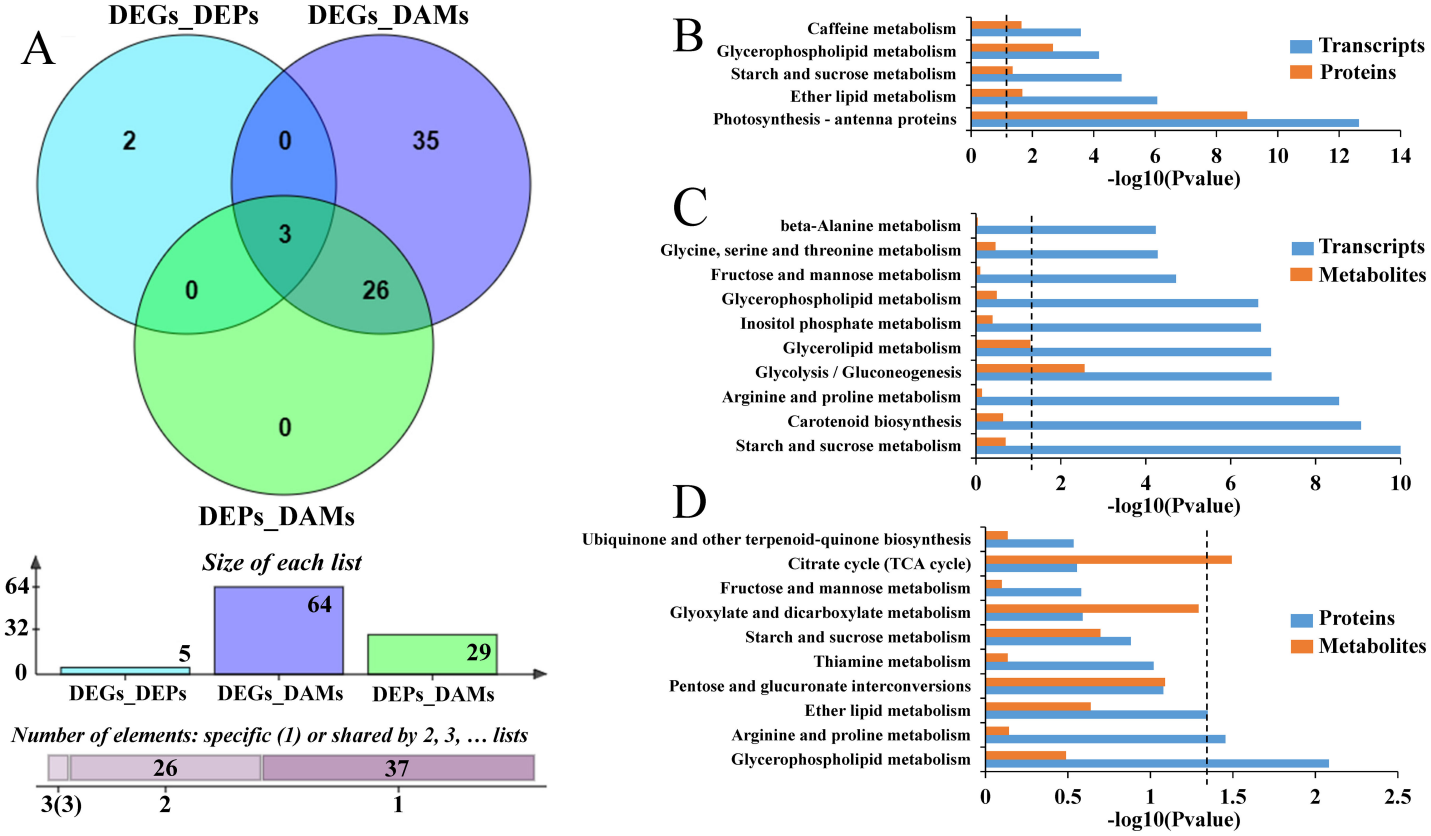
Multi-omics analysis of the common pathways for Chuanmai 104 under cold stress. **(A)** Venn diagram showing the common pathways based on differentially expressed genes (DEGs)/differentially expressed proteins (DEPs), DEGs/differentially accumulated metabolites (DAMs), and DEPs/DAMs, **(B)** The common enriched pathways based on DEGs/DEPs, **(C)** The common enriched pathways based on DEGs/DAMs, and **(D)** The common enriched pathways based on DEPs/DAMs.

To better understand the molecular mechanism associated with the tolerance of CM104 to low temperatures, we performed an integrated analysis of the multi-omics sequencing data. Comparative transcriptomic and proteomic profiling revealed DEGs with the same trend of mRNA and protein expression (**Supplementary Figure 7A**, quadrants 3 and 7) and were enriched in 17 KEGG pathways after cold stress (**Supplementary Figure 7B**). Among them, those enriched to DEPs were photosynthesis-antenna proteins, glycerophospholipid metabolism, ether lipid metabolism, caffeine metabolism, and starch and sucrose metabolism (**Figures 7A, B**; **Supplementary Figure 7C**). Integrated transcriptome and metabolome analysis revealed that DEGs and DAMs were distributed in 64 KEGG pathways, with starch and sucrose metabolism, carotenoid biosynthesis, arginine and proline metabolism, glycolysis/gluconeogenesis, and glycerolipid metabolism having the highest number of DEGs/DAPs (**Figures 7A, C**; **Supplementary Figure 8**). Proteomic and metabolomic analyses indicated the enrichment of DEPs and DAMs in 29 common KEGG pathways, with the top five pathways namely, glycerophospholipid, arginine and proline, ether lipid, pentose and glucuronate interconversions, and thiamine metabolism having the highest number of correlated DEPs/DAMs (**Figures 7A, D**).

**Figure 8:**
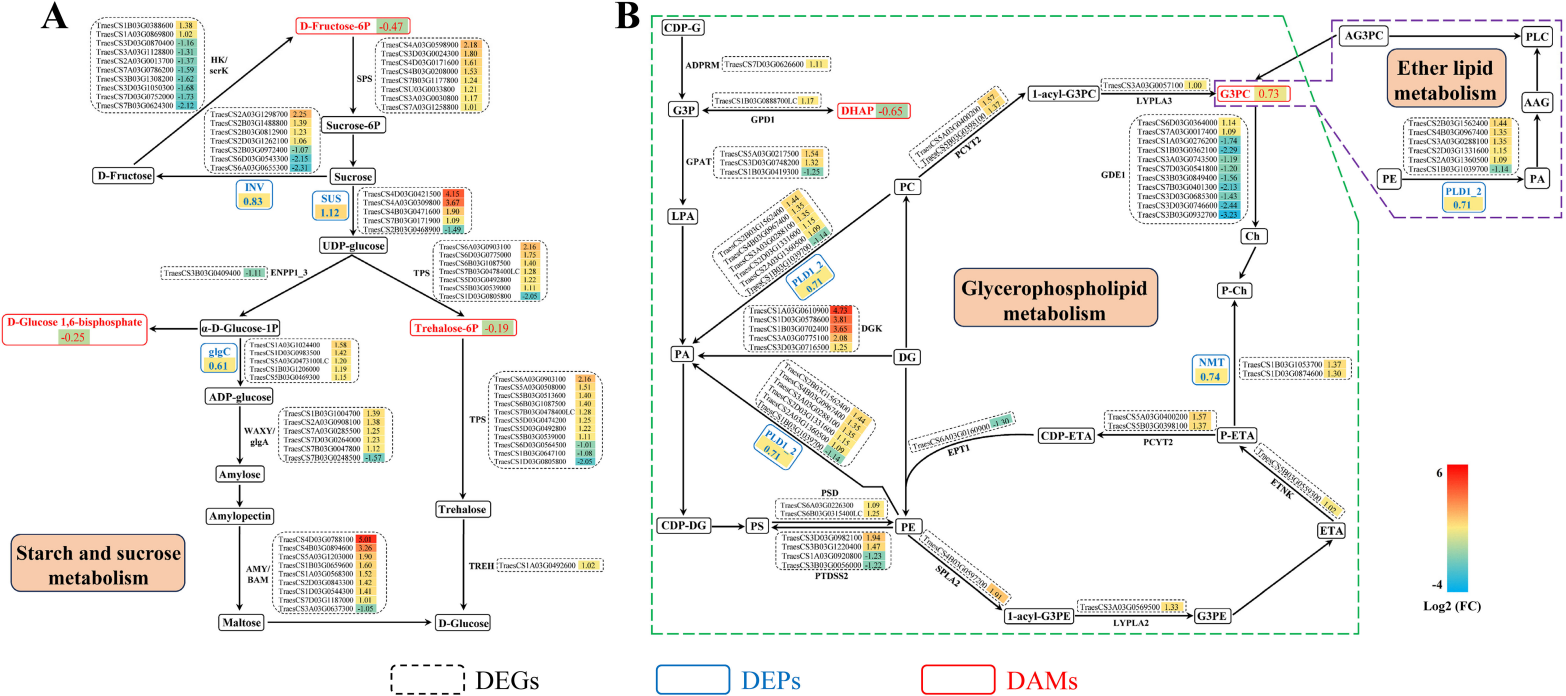
Changes of differentially expressed genes (DEGs), differentially expressed proteins (DEPs), and differentially accumulated metabolites (DAMs) involved in main metabolic pathways in Chuanmai 104 under cold stress. **(A)** Starch and sucrose metabolism. **(B)** Glycerophospholipid metabolism, and ether lipid metabolism. 1-acyl-G3PC, 1-acyl-glycerol-3-phosphatidylcholine; 1-acyl-G3PE, 1-acyl-glycerol-3-phosphatidylethanolamine; AAG, 1-alkenyl-2-acyl-glycerol; α-D-Glucose-1P, α-Glucose-1-phosphate; ADPRM, manganese-dependent ADP-ribose/CDP-alcohol diphosphatase; AG3PC, 1-alkenyl-3-phosphatidylcholine; AMY, alpha-amylase; BAM, beta-amylase; CDP-ETA, cytidine diphosphate-ethanolamine; CDP-DG, cytidine diphosphate-diacyl-glycerol; CDP-G, cytidine diphosphate-glycerol; Ch, choline; GDE1, glycerophosphodiester phosphodiesterase; D-Fructose-6P, D-Fructose 6-Phosphate; DG, diacylglycerol; DGK, diacylglycerol kinase; DHAP, dihydroxyacetone phosphate; ENPP1_3, ectonucleotide pyrophosphatase/phosphodiesterase family member 1/3; EPT1, ethanolaminephosphotransferase; ETA, ethanolamine; ETNK, ethanolamine kinase; G3P, glycerol 3-phosphate; G3PC, glycerol-3-phosphocholine; G3PE, glycerol-3-phosphoethanolamine; glcC, glucose-1-phosphate adenylyltransferase; glgA, starch synthase; GPD1, glycerol-3-phosphate dehydrogenase; GPAT, glycerol-3-phosphate acyltransferase; HK, hexokinase; INV, beta-fructofuranosidase; LPA, lysophosphatidic acid; LYPLA2, lysophospholipase II; LYPLA3, lysophospholipase III; NMT, phosphoethanolamine N-methyltransferase; PA, phosphatidic acid; PC, phosphatidylcholine; P-Ch, phospho-choline; PCYT2, ethanolamine-phosphate cytidylyltransferase; PE, phosphatidylethanolamine; P-ETA, phosphor-ethanolamine; PLC, plasmenylcholine PLD1_2, phospholipase D1/2; PS, phosphatidylserine; PSD, phosphatidylserine decarboxylase; PTDSS2, phosphatidylserine synthase 2; scrK, fructokinase; SPLA2, secretory phospholipase A2; SPS, sucrose-phosphate synthase; Sucrose-6P, Sucrose6-phosphate; SUS, sucrose synthase; TREH, alpha, alpha-trehalase; TPS, alpha, alphatrehalose-phosphate synthase; Trehalose-6P, Trehalose 6-phosphate; WAXY, granule-bound starch synthase.

Integrated analysis of the transcriptomics, proteomics, and metabolomics data sets identified significant enrichment in three common KEGG pathways (**Figure 7A**), including starch and sucrose, glycerophospholipid, and ether lipid metabolism. In the starch and sucrose metabolism pathway, most of the genes encoding sucrose-phosphate synthase (SPS, EC:2.4.1.14), sucrose synthase (SS, EC:2.4.1.13), alpha, alpha-trehalose-phosphate synthase (α,α-TPS, EC:2.4.1.12), glucose-1-phosphate adenylyltransferase (EC:2.7.7.27), α-amylase (EC:3.2.1.1) and granule-bound starch synthase (EC:2.4.1.242) were upregulated. Three metabolites, including D-Fructose 6-Phosphate, D-Glucose 1,6-bisphosphate and Trehalose 6-phosphate were downregulated, while three proteins namely, SS, glucose-1-phosphate adenylyltransferase and β-fructofuranosidase were upregulated in cold stressed wheat (**Figure 8A**). For the glycerophospholipid metabolism pathway, most of the genes encoding phospholipase D1/2 (EC:3.1.4.4), diacylglycerol kinase (EC:2.7.1.107), and phosphoethanolamine N-methyltransferase (EC:2.1.1.103) were upregulated, while most of the genes encoding glycerophosphodiester phosphodiesterase were downregulated. One metabolite, glycerol-3-phosphocholine was upregulated and another, dihydroxyacetone phosphate was downregulated, while all the two proteins, namely phospholipase D1/2 and phosphoethanolamine N-methyltransferase were upregulated in wheat exposed to cold (**Figure 8B**). In addition, the metabolite choline alfoscerate, and the gene encoding phospholipase D1/2 and its corresponding protein were implicated in the ether lipid metabolism pathway (**Figure 8B**).

## 4 Discussion

### 4.1 Agronomic and physiological response to cold stress at the booting stage

The booting stage is the key stage in the differentiation and development of wheat spikes and is easily influenced by external temperature (Yu et al., 2022). Extreme low-temperature stress adversely impacts anther development, subsequently reducing seed setting rates, grain numbers per spike and yields (Liu et al., 2020). Low temperature also causes abnormal meiosis of pollen mother cells, delayed disintegration of the tapetal layer, and insufficient accumulation of anther starch grains, resulting in pollen abortion (Barton et al., 2014). In this research, pollen viability and seed set were significantly affected by cold stress in both varieties, with more severe effects in those with longer exposure to low temperatures. Chakrabarti et al. (2011) found a significant positive correlation (*R* ^2^ = 0.65) between pollen germination percentage and seed setting rate in wheat crops, consistent with the findings of this experiment. The cold-tolerant variety CM104 exhibited a significantly lower pollen abortion rate than the cold-sensitive variety CM42 across all the treatments, thereby maintaining a high seed setting rate and mitigating the impact of cold stress on its yields.

Crop plants experience various physiological changes when exposed to low-temperature stress and the strength of cold tolerance in wheat is closely linked to changes in different osmoregulatory substances, hormone contents and protective enzyme systems (Thakura et al., 2010). The response to cold stress in different types of wheat is found to be divergent. Winter wheat requires a low temperature during the vernalization period and can survive cold conditions during the early vegetative stages (Ejaz et al., 2023). Compare to spring wheat, winter wheat showed higher cold tolerance, potentially due to their effective osmoregulation ability, high photosynthetic capacity, and lower lipid peroxidation under low-temperature conditions (Zhang et al., 2016; Hassan et al., 2021). As the main osmoregulatory substances, the increase in soluble sugars and proteins and proline in cold-stressed plants effectively increases the cytosol concentrations and lowers the freezing point of the cells, aiding in maintaining the integrity of the cell membrane (Chen et al., 2014). In our study, CM104 had higher levels of soluble sugars, soluble proteins and proline than CM42 under various cold treatments, suggesting that CM104 synthesizes more osmoregulatory substances to enhance its cold tolerance and mitigate cold damage. Cold stress also alters the hormonal balance in plants by affecting levels of ABA and GA_3_ (Hassan et al., 2021; Soualiou et al., 2022). In this study, cold stress increased ABA but decreased GA_3_ content in young spikes of both varieties. The ABA content was higher in CM104 than in CM42, whereas the GA_3_ content was lower, which was conducive to promoting stomatal closure to maintain water balance and increase or maintain the content of chlorophyll, proteins and nucleic acids, thereby increasing the cold tolerance. The low-temperature stress induces excessive reactive oxygen species (ROS) in plants, disrupting cellular balance and causing severe cell membrane damage and lipid peroxidation. Therefore, plants have evolved a sophisticated enzyme system, including SOD, POD, and CAT, to mitigate ROS-induced plasma membrane damage (Zhang et al., 2016; Hail et al., 2017; Jiang et al., 2022). In our current study, CM104 exhibited higher antioxidant enzyme activities than CM42 under various cold treatments, suggesting its greater efficiency in ROS scavenging and reduced cold-induced damage in CM104 than CM42.

### 4.2 Cold tolerance differences between the two varieties relate to differentially expressed TF genes

In crop plants, cold stress usually causes significant changes in gene expression patterns and cold tolerance is closely linked to the co-expression of these genes (Gusain et al., 2023). The TFs are crucial in plant development and environmental stress resistance, and various TF families, including AP2/ERF, bHLH, MYB, HSF, NAC, bZIP, and WRKY, play important roles in cold-responsive gene expression (Chen et al., 2014; Ahad et al., 2023). We identified more TFs in CM104 than in CM42, with the most differentially expressed TF genes in both cultivars belonging to bHLH, AP2/ERF, and HSF families. The AP2/ERFs are a significant TF family in plants, and are integral to plant growth, development, stress responses and biosynthetic pathways (Singh et al., 2002). The CBFs, which are members of the AP2/ERF family, regulate a series of cold-regulated (COR) genes as has been shown in the connection between *CBF3* and the frost-tolerance locus *Fr-A2*, demonstrating that *CBF3* positively regulates cold stress responses in wheat (Vágújfalvi et al., 2003). The *TaCBF14* and *TaCBF15* genes which enhance low-temperature tolerance in spring barley have also been identified in winter wheat (Solteísz et al., 2013), further supporting the roles of CBFs in regulating cold stress. The overexpression of ERF TFs, *TERF2/LeERF2* significantly enhanced the cold tolerance in tomato and tobacco (Zhang and Huang, 2010), while *TaPIE1*, an ERF gene family activated the defense and ethylene signalling pathways in wheat, thereby improving its tolerance to cold stress (Zhu et al., 2014). In our study, all the ERF family DEGs were up-regulated in CM104, while some were down-regulated in CM42, indicating that these TFs may be crucial in CM104’s cold tolerance mechanism.

The bHLH family genes are the second largest TFs in plants and are crucial in plant stress response. Recent studies have identified numerous bHLH TFs that improve plant cold tolerance via the CBF-COR pathway or by enhancing ROS scavenging (Singh et al., 2002). Zheng et al. (2021) found that the apple bHLH homolog *MdPIF3* negatively regulates cold tolerance. However, Yang et al. (2022) demonstrated that *NtbHLH123* enhances cold stress in tobacco by regulating pathways and maintaining reactive oxygen homeostasis. In wheat, the overexpression of the bHLH gene *TaMYC2* improved cold tolerance in *Arabidopsis* by interacting with *TaICE41* to regulate the ICE-CBF-COR module (Wang et al., 2022). As important regulatory factors, HSFs play significant roles in stress responses to various biotic and abiotic stresses in wheat and other plants (Singh et al., 2002). For instance, *HSFA1b* enhances heat tolerance by activating OPR3 expression and promoting JA synthesis in wheat and *Arabidopsis* (Tian et al., 2020), while the overexpression of the wheat HSF genes, *TaHsf3* and *TaHsfA6f* in *Arabidopsis* improves tolerance to heat, cold, and drought stress (Zhang et al., 2013; Bi et al., 2020). In this study, some bHLH and HSF TFs were up-regulated while others were down-regulated under cold stress in both varieties, indicating their complex response mechanism to low temperatures in wheat. In addition, other TFs such as the CSA and MTB2 TFs, which were uniquely identified in CM104, may be directly related to its cold tolerance but deserve further analysis in future studies.

### 4.3 Regulation of hormone signal transduction for cold response in wheat

Plant hormones are crucial in managing abiotic stress by activating cold response genes and coordinating various signalling pathways (Frascaroli, 2018; Gusain et al., 2023). In this study, we found genes related to phytohormone activities such as ABA in response to cold stress in wheat. A metabolomic analysis demonstrated significantly elevated ABA and Ja-L-Ile levels in wheat under cold stress and identified critical pathways linked to ABA/JA signalling as significant regulators of cold tolerance in wheat (Zhao et al., 2019). In addition, Abhinandan et al. (2018) showed the performance of essential functions in various abiotic stresses by the PP2C gene family, which is also a core component in the ABA-dependent signalling pathways. All DEGs related to PP2C were upregulated in the CM104 and CM42 under cold stress. However, the PP2C DEGs namely TraesCS1A03G1005000, TraesCS2A03G0000700 and TraesCS3A03G0542200) were unique to CM104, most likely due to the ABA-induced expression of PP2C, which regulates SnRK2 protein kinase activity to initiate ABA signalling (Zhao et al., 2019; Jiang et al., 2022). Therefore, the variation in the expression of PP2C between the two wheat cultivars indicates its potential significance in cold tolerance.

Auxin, a key regulator of plant growth and development, has an unclear role in response to abiotic stresses like cold (Abhinandan et al., 2018). It interacts with ABA and JA to regulate the expression of TFs, such as WRKYs, thereby enhancing cold tolerance in plants (Raza et al., 2023). The wheat auxin response factor (ARF) genes are also crucial in the interaction between abiotic stress responses and the ABA signalling pathway (Xu et al., 2020), while the auxin-responsive protein gene (IAA14) may play a role in wheat cold tolerance (Jiang et al., 2022). We annotated TraesCS6B03G0760600 and TraesCS6A03G0612400 as ARF8 and TraesCS5D03G0986600 as IAA14, and showed their upregulation in both cultivars, indicating their potential role in wheat cold tolerance. However, there is a need to study the role and function of auxin under cold stress in wheat.

Ethylene negatively regulates plant growth and influences various physio-biochemical mechanisms associated with biotic and abiotic stresses (Soualiou et al., 2022). For instance, ethylene-induced cold stress tolerance activates defense-related genes and regulates ROS-scavenging pathways via signalling cascades to mitigate the effects of cold stress (Raza et al., 2023). The upregulation of ethylene response factor (ERF) genes, *TaERF1* and *TaERF3* enhances tolerance to salt, drought, and cold in wheat (Abhinandan et al., 2018). In the present study, most DEGs related to ERF were upregulated in CM104 and downregulated in CM42, most likely due to the differences in cold resistance of the two varieties.

### 4.4 Important proteins and metabolites associated with the cold stress response in wheat

During cold stress in plants, a series of response gene expression products or cold-responsive proteins are produced via signal transduction to mitigate low-temperature damage and enhance cold tolerance (Frascaroli, 2018; Ahad et al., 2023). The LEA/dehydrin proteins are the key types of cold-responsive proteins, crucial in plant response to cold stress (Vítámvás et al., 2010; Kosová et al., 2021; Soualiou et al., 2022; Xu et al., 2022). For instance, the overexpression of maize dehydrin gene *ZmDHN2b* in tobacco enhances cold tolerance by decreasing malondialdehyde accumulation and electrolyte leakage (Xing et al., 2011), while the overexpression of *Solanum habrochaites* dehydrin gene *ShDHN* in tomato plants enhances its adaptation to cold stress by improving ROS scavenging and water-retention capacities (Liu et al., 2015). In wheat, the overexpression of the dehydrin gene *Wdhn13* in *Arabidopsis* enhances freezing stress tolerance, while gene silencing increases its sensitivity to cold (Xu et al., 2022). Vítámvás et al. (2019) found a significant correlation between dehydrin transcript/protein accumulation and winter survival in both winter wheat and winter barley plants in the field conditions. In this study, many dehydrin genes and proteins such as TraesCS6A03G0900100 and TraesCS6B03G1082300 were highly up-regulated in CM104, indicating that they may improve cold tolerance in this variety. Future investigations are also needed to comprehensively explore the function of these important cold-responsive proteins in wheat.

In addition to genes and proteins, numerous metabolites such as p-coumaroyl regulate plant growth and development and confer cold stress resistance in plants (Lv et al., 2022). Other metabolites, such as sucrose, phenylalanine, glutamine, glutamate and proline enhance cold acclimation in tobacco (Song et al., 2024). In this study, cold stress significantly upregulated lysoPC 22:6 and 5-L-glutamyl-L-amino acid in wheat. LysoPC occurs in the cell membrane, with a functional role in its bilayer. The LysoPC level is often adjusted by lipid remodeling, which is crucial in cold stress tolerance in maize (Gao et al., 2024). On the other hand, 5-L-glutamyl-L-amino acid is linked to glutathione metabolism, a crucial pathway for oxidative stress defense and its increased levels aid in defense against high saline-alkali stress in rice leaves (Qian et al., 2023). Therefore, the significantly increased levels of LysoPC 22:6 and 5-L-Glutamyl-L-amino acid indicate their potential importance in cold tolerance in wheat.

### 4.5 Key metabolic pathways involved in the cold tolerance of CM104

Cold acclimation, deacclimation and reacclimation are complex, evolution-based physiological processes through which plants respond to their environment. These processes all involve multiple genes and metabolic pathways (Kosová et al., 2025). Integrated multi-omics analysis could provide comprehensive information on key metabolic pathways involved in cold stress (Yadav, 2010; McLoughlin et al., 2018). Our comprehensive analysis of the transcriptome, proteome and metabolome, showed that several associated DEGs, DEPs and DAMs were involved in starch and sucrose, glycerophospholipid and ether lipid metabolism. Starch and sucrose metabolism is a crucial plant metabolic pathway that significantly influences responses to abiotic stress (Chen et al., 2014) and may be closely related to cold resistance in wheat (Zhao et al., 2019; Jiang et al., 2022; Li et al., 2023). Soluble sugars, such as sucrose, glucose, trehalose and maltose involved in this pathway provide energy that enhances antioxidative capacity, maintains membrane stability, and mitigates cold stress damage (Zhang et al., 2016; Lv et al., 2022).

Our study demonstrated a significant increase in soluble sugars in both varieties under cold stress. Similarly, DEPs named SS and β-fructofuranosidase, and most DEGs encoding SPS, SS, α,α-TPS, and α-amylase associated with the regulation of sucrose, trehalose and maltose production, were highly upregulated. SS and β-fructofuranosidase hydrolyze sucrose to increase levels of glucose, which is crucial for osmotic protection and energy production during cold stress. In addition, the D-fructose 6-phosphate and trehalose 6-phosphate metabolites were downregulated, indicating that low temperatures might inhibit their synthesis or cause their decomposition into downstream substances of sucrose and trehalose. These results show that the metabolism of starch and sucrose improved, contributing to cold tolerance in CM104 at the booting stage.

The metabolic pathways associated with membrane lipids tend to change significantly under cold stress, since plant membranes are the first barrier to respond to external environmental stimuli (Chen et al., 2014; Hou et al., 2016). In the present study, the genes and proteins encoding phospholipase D1/2 and phosphoethanolamine N-methyltransferase, and the metabolite of glycerol-3-phosphocholine related to glycerophospholipid and ether lipid metabolism pathway were upregulated. These genes, proteins and metabolites are involved in plasma membrane synthesis and degradation. Phospholipase D is a crucial enzyme that plays a significant role in abiotic and biotic stress signalling in plants. It mediates plant defense responses mainly by participating in resistance signalling pathways associated with ROS (Wang, 2005). Phosphoethanolamine N-methyltransferase catalyzes the methylation of phosphoethanolamine to phosphocholine, which plays a significant role in abiotic stress response. Phosphoethanolamine N-methyltransferase transcript and its corresponding protein were strongly upregulated by low temperature in wheat (Charron et al., 2002), consistent with our findings. This indicates the important role of phospholipase D1/2 and phosphoethanolamine N-methyltransferase in response to cold in wheat. Besides, glycerol-3-phosphocholine is a potential biomarker for drought stress responses and also serves as an important nutrient source (Chen et al., 2022). In our study, glycerol-3-phosphocholine was upregulated, indicating its significant role in response to cold in wheat. Overall, our findings identified potential gene, protein, and metabolite candidates linked to metabolic pathways involved in cold responses during the booting stage in wheat. However, further research is required to confirm the specific functions of these genes, proteins, and metabolites, and to integrate them into future wheat breeding programs to aid in the selection and breeding of cold-tolerant wheat varieties.

## 5 Conclusions

**Figure 9:**
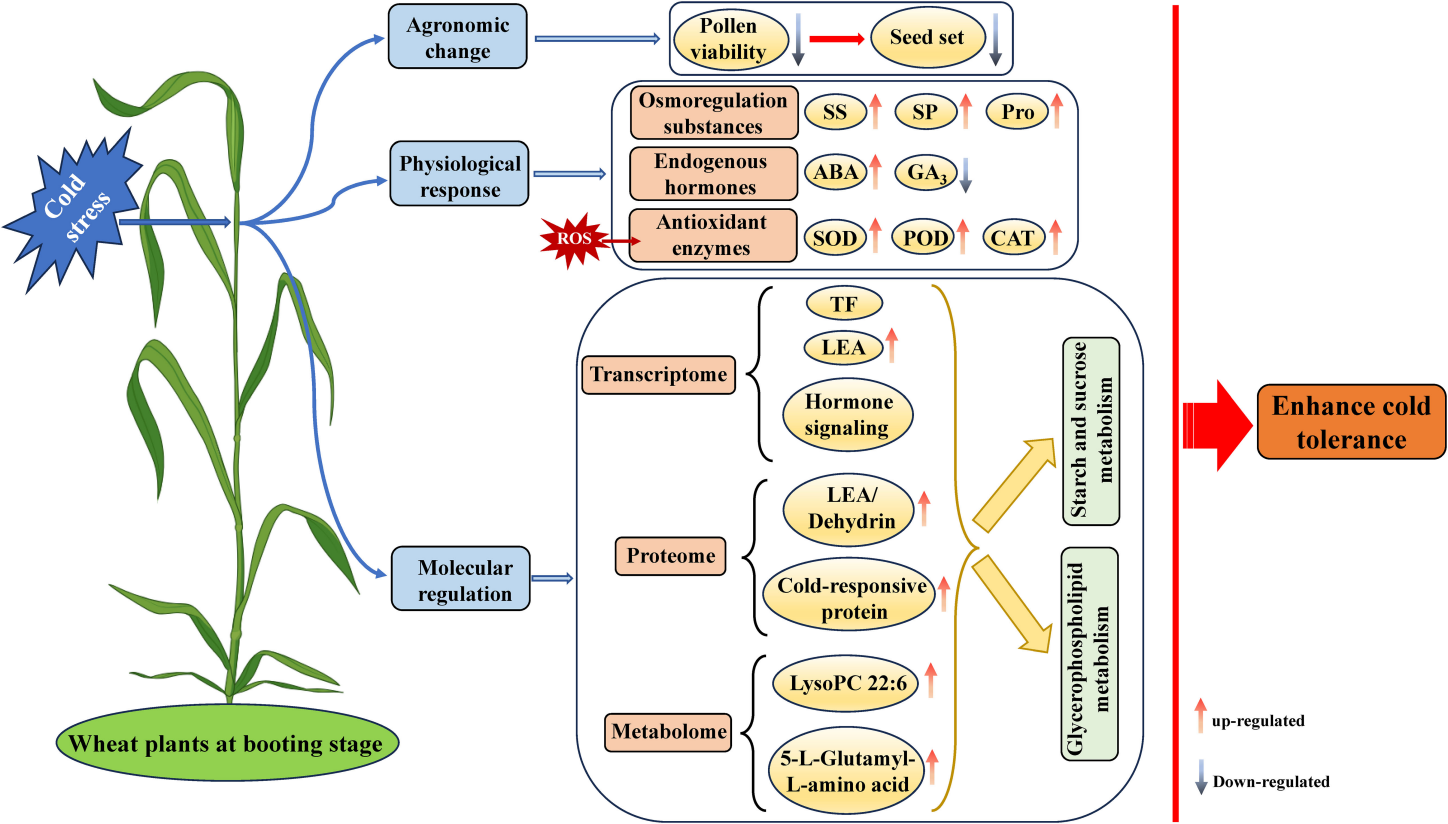
The working model for wheat plants response to cold stress at the booting stage. ABA, abscisic acid; CAT, catalase; GA_3_, gibberellin; LEA, late embryogenesis abundant; Pro, proline; POD, peroxidase; ROS, reactive oxygen species; SOD, superoxide dismutase; SP, soluble protein; SS, soluble sugar; TF, transcription factor.

This study utilized a multi-omics approach together with physiological perspectives to elucidate the low-temperature response mechanism in wheat during the booting stage. A working model for wheat plants response to cold stress at the booting stage is proposed (**Figure 9**). The cold stress severely affected pollen viability and seed setting rates in wheat, but increased its osmoregulatory substances and antioxidant enzymes, and revealed many key DEGs, such as TFs, LEA and hormone signalling transduction associated with cold response and tolerance. Proteomic and metabolomic analysis identified some specific DEPs, LEA/dehydrin and other cold-responsive proteins and DAMs, such as LysoPC 22:6 and 5-L-Glutamyl-L-amino acid, potentially associated with cold tolerance in wheat. Further integrated analysis of transcriptome, proteome, and metabolome datasets identified starch and sucrose, and glycerophospholipid metabolism as the key pathways for cold tolerance in wheat. Overall, genes, proteins and metabolites identified in this study can potentially aid in the selection and breeding of cold-tolerant wheat varieties.

## Data Availability

The datasets presented in this study can be found in online repositories. The raw transcriptomic data in this study have been deposited in Sequence Read Archive (SRA) database of the National Center for Biotechnology Information (NCBI) under BioProject ID PRJNA1131989. The mass spectrometry proteomics data have been deposited to the ProteomeXchange Consortium via the iProX repository (https://www.iprox.cn/) with the dataset identifier PXD053970.

## References

[B1] Abhinandan K. Skori L. Stanic M. Hickerson N. M. Jamshed M. Samuel M. A. (2018). Abiotic stress signaling in wheat–an inclusive overview of hormonal interactions during abiotic stress responses in wheat. Front. Plant Sci. 9. doi: 10.3389/fpls.2018.00734, PMID: 29942321 PMC6004395

[B2] Ahad A. Gul A. Batool T. S. Huda N. U. Naseeer F. Abdul Salam U.. (2023). Molecular and genetic perspectives of cold tolerance in wheat. Mol. Biol. Rep. 50, 6997–7015. doi: 10.1007/s11033-023-08584-1, PMID: 37378744

[B3] Barton D. A. Cantrill L. C. Law A. M. Phillips C. G. Sutton B. G. Overall R. L. (2014). Chilling to zero degrees disrupts pollen formation but not meiotic microtubule arrays in *Triticum aestivum* L. Plant Cell Environ. 37, 2781–2794. doi: 10.1111/pce.12358, PMID: 24762030

[B4] Bi H. Zhao Y. Li H. Liu W. (2020). Wheat heat shock factor TaHsfA6f increases ABA levels and enhances tolerance to multiple abiotic stresses in transgenic plants. Int. J. Mol. Sci. 21, 3121. doi: 10.3390/ijms21093121, PMID: 32354160 PMC7247712

[B5] Chakrabarti B. Singh S. D. Nagarajan S. Aggarwal P. K. (2011). Impact of temperature on phenology and pollen sterility of wheat varieties. Aust. J. Crop Sci. 5, 1039–1043. doi: 10.1007/s40502-013-0002-6

[B6] Charron J. B. F. Breton G. Danyluk J. Muzac I. Ibrahim R. K. Sarhan F. (2002). Molecular and biochemical characterization of a cold-regulated phosphoethanolamine N-methyltransferase from wheat. Plant Physiol. 129, 363–373. doi: 10.1104/pp.001776, PMID: 12011366 PMC155899

[B7] Chen L. Shen Y. Yang W. Pan Q. Li C. Sun Q.. (2022). Comparative metabolic study of two contrasting Chinese cabbage genotypes under mild and severe drought stress. Int. J. Mol. Sci. 23, 5947. doi: 10.3390/ijms23115947, PMID: 35682623 PMC9180449

[B8] Chen L. Xiang H. Miao Y. Zhang L. Guo Z. Zhao X.. (2014). An overview of cold resistance in plants. J. Agron. Crop Sci. 200, 237–245. doi: 10.1111/jac.12082

[B9] Ejaz I. Pu X. Naseer M. A. Bohoussou Y. N. D. Liu Y. Farooq M.. (2023). Cold and drought stresses in wheat: A global meta-analysis of 21st century. J. Plant Growth Regul. 42, 5379–5395. doi: 10.1007/s00344-023-10960-x

[B10] Fábián A. Sáfrán E. Szabó-Eitel G. Barnabás B. Jäger K. (2019). Stigma functionality and fertility are reduced by heat and drought co-stress in wheat. Front. Plant Sci. 10. doi: 10.3389/fpls.2019.00244, PMID: 30899270 PMC6417369

[B11] FAO (Food and Agriculture Organization) (2022). FAOSTAT Database. Available online at: http://www.fao.org/faostat/.

[B12] Frascaroli E. (2018). Breeding Cold-Tolerant Crops, in Cold Tolerance in Plants: Physiological, Molecular and Genetic Perspectives. Eds. Wani V. Herath S. H. (Cham: Springer International Publishing), 159–177. doi: 10.1007/978-3-030-01415-5_9

[B13] Gao L. Jiang H. Li M. Wang D. Xang H. Zeng R.. (2024). Genetic and lipidomic analyses reveal the key role of lipid metabolism for cold tolerance in maize. J. Genet. Genomics 51, 326–337. doi: 10.1016/j.jgg.2023.07.004, PMID: 37481121

[B14] Gusain S. Joshi S. Joshi R. (2023). Sensing, signalling, and regulatory mechanism of cold-stress tolerance in plants. Plant Physiol. Bioch. 197, 107646. doi: 10.1016/j.plaphy.2023.107646, PMID: 36958153

[B15] Hail Z. R. Mohammed A.-I. Michael P. F. (2017). Advances in physiological and molecular aspects of plant cold tolerance. J. Plant Interact. 12, 143–157. doi: 10.1080/17429145.2017.1308568

[B16] Hassan M. A. Xiang C. Farooq M. Muhammad N. Yan Z. Hui X.. (2021). Cold stress in wheat: plant acclimation responses and management strategies. Front. Plant Sci. 12. doi: 10.3389/fpls.2021.676884, PMID: 34305976 PMC8299469

[B17] Hou Q. Ufer G. Bartels D. (2016). Lipid signalling in plant responses to abiotic stress. Plant Cell Environ. 39, 1029–1048. doi: 10.1111/pce.12666, PMID: 26510494

[B18] Ji H. Xiao L. Xia Y. Song H. Liu B. Tang L.. (2017). Effects of jointing and booting low temperature stresses on grain yield and yield components in wheat. Agric. For. Meteorol. 243, 33–42. doi: 10.1016/j.agrformet.2017.04.016

[B19] Jiang G. Hassan M. A. Muhammad N. Arshad M. Chen X. Xu Y.. (2022). Comparative physiology and transcriptome analysis of young spikes in response to late spring coldness in wheat (*Triticum aestivum* L.). Front. Plant Sci. 13. doi: 10.3389/fpls.2022.811884, PMID: 35185984 PMC8850991

[B20] Jorrin-Novo J. V. (2014). Plant proteomics methods and protocols. Methods Mol. Biol. 1072, 3–13. doi: 10.1007/978-1-62703-631-3_1, PMID: 24136510

[B21] Kosová K. Klíma M. Prášil I. T. Vítámvás P. (2021). COR/LEA proteins as indicators of frost tolerance in Triticeae: A comparison of controlled versus field conditions. Plants 10, 789. doi: 10.3390/plants10040789, PMID: 33923804 PMC8073581

[B22] Kosová K. Nešporová T. Vítámvás P. Vítámvás J. Klíma M. Ovesná J.. (2025). How to survive mild winters: Cold acclimation, deacclimation, and reacclimation in winter wheat and barley. Plant Physiol. Bioch 220, 109541. doi: 10.1016/j.plaphy.2025.109541, PMID: 39862458

[B23] Li L. Han C. Yang J. Tian Z. Jiang R. Yang F.. (2023). Comprehensive transcriptome analysis of responses during cold stress in wheat (*Triticum aestivum* L.). Genes 14, 844. doi: 10.3390/genes14040844, PMID: 37107602 PMC10137996

[B24] Li X. Jiang D. Liu F. (2016). Winter soil warming exacerbates the impacts of spring low temperature stress on wheat. J. Agron. Crop Sci. 202, 554–563. doi: 10.1111/jac.12177

[B25] Limin A. E. Fowler D. B. (2006). Low-temperature tolerance and genetic potential in wheat (*Triticum aestivum* L.): response to photoperiod, vernalization, and plant development. Planta 224, 360–366. doi: 10.1007/s00425-006-0219-y, PMID: 16440213

[B26] Liu H. Yu C. Li H. Ouyang B. Wang T. Zhang J.. (2015). Overexpression of *ShDHN*, a dehydrin gene from *Solanum habrochaites* enhances tolerance to multiple abiotic stresses in tomato. Plant Sci. 231, 198–211. doi: 10.1016/j.plantsci.2014.12.006, PMID: 25576005

[B27] Liu L. Xia Y. Liu B. Chang C. Xiao L. Shen J.. (2020). Individual and combined effects of jointing and booting low-temperature stress on wheat yield. Eur. J. Agron. 113, 125989. doi: 10.1016/j.eja.2019.125989

[B28] Liu M. Tong H. Liu Y. Li C. Wu X. Li M.. (2021). Genetic progress in grain yield and the associated physiological traits of popular wheat in southwestern China from 1969 to 2012. Crop Sci. 61, 1971–1986. doi: 10.1002/csc2.20448

[B29] Liu L. Xu H. Liu S. Liu X. (2023a). China’s response to extreme weather events must be long term. Nat. Food 4, 1022–1023. doi: 10.1038/s43016-023-00892-w, PMID: 38030889

[B30] Liu X. Renzengwangdui Tang S. Zhu Y. Wang M. Cao B.. (2023b). Metabolomic analysis and antibacterial and antioxidant activities of three species of *Artemisia* plants in Tibet. BMC Plant Biol. 23, 208. doi: 10.1186/s12870-023-04219-6, PMID: 37081377 PMC10120219

[B31] Livak K. J. Schmittgen T. D. (2001). Analysis of relative gene expression data using real-time quantitative PCR and the 2^^-△△Ct^ method. Methods 25, 402–408. doi: 10.1006/meth.2001.1262, PMID: 11846609

[B32] Lv L. Dong C. Liu Y. Zhao A. Zhang Y. Li H.. (2022). Transcription-associated metabolomic profiling reveals the critical role of frost tolerance in wheat. BMC Plant Biol. 22, 1–22. doi: 10.1186/s12870-022-03718-2, PMID: 35820806 PMC9275158

[B33] McLoughlin F. Augustine R. C. Marshall R. S. Li F. Q. Kirkpatrick L. D. Otegui M. S.. (2018). Maize multi-omics reveal roles for autophagic recycling in proteome remodeling and lipid turnover. Nat. Plants 4, 1056–1070. doi: 10.1038/s41477-018-0299-2, PMID: 30478358

[B34] Qian G. Wang M. Wang X. Liu K. Li Y. Bu Y.. (2023). Integrated transcriptome and metabolome analysis of rice leaves response to high saline–alkali stress. Int. J. Mol. Sci. 24, 4062. doi: 10.3390/ijms24044062, PMID: 36835473 PMC9960601

[B35] Raza A. Charagh S. Najafi-Kakavand S. Abbas S. Shoaib Y. Anwar S.. (2023). Role of phytohormones in regulating cold stress tolerance: physiological and molecular approaches for developing cold-smart crop plants. Plant Stress 8, 100152. doi: 10.1016/j.stress.2023.100152

[B36] Singh K. Foley R. Oñate-Sánchez L. (2002). Transcription factors in plant defense and stress responses. Curr. Opin. Plant Biol. 5, 430–436. doi: 10.1016/S1369-5266(02)00289-3, PMID: 12183182

[B37] Solteísz A. Smedley M. Vashegyi I. Galiba G. Harwood W. Vaíguíjfalvi A. (2013). Transgenic barley lines prove the involvement of TaCBF14 and *TaCBF15* in the cold acclimation process and in frost tolerance. J. Exp. Bot. 64, 1849–1862. doi: 10.1093/jxb/ert050, PMID: 23567863 PMC3638819

[B38] Song X. Wang H. Wang Y. Zeng Q. Zheng X. (2024). Metabolomics combined with physiology and transcriptomics reveal how *Nicotiana tabacum* leaves respond to cold stress. Plant Physiol. Bioch. 208, 108464. doi: 10.1016/j.plaphy.2024.108464, PMID: 38442629

[B39] Soualiou S. Duan F. Li X. Zhou W. (2022). Crop production under cold stress: An understanding of plant responses, acclimation processes, and management strategies. Plant Physiol. Bioch. 190, 47–61. doi: 10.1016/j.plaphy.2022.08.024, PMID: 36099808

[B40] Thakura P. Kumara S. Malika J. A. Bergerb J. D. Nayyara H. (2010). Cold stress effects on reproductive development in grain crops: an overview. Environ. Exp. Bot. 67, 429–443. doi: 10.1016/j.envexpbot.2009.09.004

[B41] Tian X. Wang F. Zhao Y. Lan T. Yu K. Zhang L.. (2020). Heat shock transcription factor A1b regulates heat tolerance in wheat and Arabidopsis through OPR3 and jasmonate signalling pathway. Plant Biotechnol. J. 18, 1109–1111. doi: 10.1111/pbi.13268, PMID: 31559685 PMC7152600

[B42] Vágújfalvi A. Galiba G. Cattivelli L. Dubcovsky J. (2003). The cold-regulated transcriptional activator *Cbf3* is linked to the frost-tolerance locus *Fr-A2* on wheat chromosome 5A. Mol. Gen. Genomics 269, 60–67. doi: 10.1007/s00438-003-0806-6, PMID: 12715154 PMC4743881

[B43] Vítámvás P. Kosová K. Musilová J. Holková L. Mařík P. Smutná P.. (2019). Relationship between dehydrin accumulation and winter survival in winter wheat and barley grown in the field. Front. Plant Sci. 10. doi: 10.3389/fpls.2019.00007, PMID: 30761163 PMC6361858

[B44] Vítámvás P. Kosová K. Prášilová P. Prášil I. T. (2010). Accumulation of WCS120 protein in wheat cultivars grown at 9 C or 17 C in relation to their winter survival. Plant Breed. 129, 611–616. doi: 10.1111/j.1439-0523.2010.01783.x

[B45] Wang X. (2005). Regulatory functions of phospholipase D and phosphatidic acid in plant growth, development, and stress responses. Plant Physiol. 139, 566–573. doi: 10.1104/pp.105.068809, PMID: 16219918 PMC1255977

[B46] Wang R. Yu M. Xoa J. Xing J. Fan X. Xu Q.. (2022). Overexpression of *TaMYC2* confers freeze tolerance by ICE-CBF-COR module in *Arabidopsis thaliana* . Front. Plant Sci. 13. doi: 10.3389/fpls.2022.1042889, PMID: 36466238 PMC9710523

[B47] Xing X. Liu Y. Kong X. Liu Y. Li D. (2011). Overexpression of a maize dehydrin gene, *ZmDHN2b*, in tobacco enhances tolerance to low temperature. Plant Growth Regul. 65, 109–118. doi: 10.1007/s10725-011-9580-3

[B48] Xu L. Wang D. Liu S. Fang Z. Su S. Guo C.. (2020). Comprehensive atlas of wheat (*Triticum aestivum L.*) AUXIN RESPONSE FACTOR expression during male reproductive development and abiotic stress. Front. Plant Sci. 11. doi: 10.3389/fpls.2020.586144, PMID: 33101350 PMC7554351

[B49] Xu K. Zhao Y. Gu J. Zhou M. Gao L. Sun R.. (2022). Proteomic analysis reveals the molecular mechanism underlying the cold acclimation and freezing tolerance of wheat (*Triticum aestivum* L.). Plant Sci. 318, 111242. doi: 10.1016/j.plantsci.2022.111242, PMID: 35351310

[B50] Yadav S. K. (2010). Cold stress tolerance mechanisms in plants. A review. Agron. Sustain. Dev. 30, 515–527. doi: 10.1051/agro/2009050

[B51] Yang X. Luo Y. Bai H. Li X. Tang S. Liao X.. (2022). DgMYB2 improves cold resistance in chrysanthemum by directly targeting DgGPX1. Hortic. Res. 9, uhab028. doi: 10.1093/hr/uhab028, PMID: 35039835 PMC8801720

[B52] Yu X. Jiang Y. Yao H. Ran L. Zang Y. Xiong F. (2022). Cytological and molecular characteristics of delayed spike development in wheat under low temperature in early spring. Crop J. 10, 840–852. doi: 10.1016/j.cj.2021.08.008

[B53] Zhang Z. Huang R. (2010). Enhanced tolerance to freezing in tobacco and tomato overexpressing transcription factor TERF2/LeERF2 is modulated by ethylene biosynthesis. Plant Mol. Biol. 73, 241–249. doi: 10.1016/j.cj.2019.09.002, PMID: 20135196

[B54] Zhang B. Jia D. Gao Z. Dong Q. He L. (2016). Physiological responses to low temperature in spring and winter wheat varieties. J. Sci. Food Agr. 96, 1967–1973. doi: 10.1002/jsfa.7306, PMID: 26095741

[B55] Zhang S. Xu Z. Li P. Yang L. Wei Y. Chen M.. (2013). Overexpression of *TaHSF3* in transgenic *Arabidopsis* enhances tolerance to extreme temperatures. Plant Mol. Biol. Rep. 31, 688–697. doi: 10.1007/s11105-012-0546-z

[B56] Zhao Y. Zhou M. Xu K. Li J. Li S. Zhang S.. (2019). Integrated transcriptomics and metabolomics analyses provide insights into cold stress response in wheat. Crop J. 7, 857–866. doi: 10.1016/j.cj.2019.09.002

[B57] Zheng P. Yang Y. Zhang S. You C. Zhang Z. Hao Y. (2021). Identification and functional characterization of *MdPIF3* in response to cold and drought stress in *Malus domestica* . Plant Cell Tiss. Org. 144, 435–447. doi: 10.1007/s11240-020-01968-2

[B58] Zheng C. Zhu Y. Wang C. Guo T. (2016). Wheat grain yield increase in response to pre-anthesis foliar application of 6-benzylaminopurine is dependent on floret development. PloS One 11, e0156627. doi: 10.1371/journal.pone.0156627, PMID: 27258059 PMC4892633

[B59] Zhu X. Qi L. Liu X. Cai S. Xu H. Huang R.. (2014). The wheat ethylene response factor transcription factor pathogen-induced ERF1 mediates host responses to both the necrotrophic pathogen *Rhizoctonia cerealis* and freezing stresses. Plant Physiol. 164, 1499–1514. doi: 10.1104/pp.113.229575, PMID: 24424323 PMC3938636

