# Supplementary material for: Multi-omics analysis reveals the physiological and molecular response to cold stress in different spring wheat cultivars at the booting stage
Source: Front Plant Sci. 2025 Aug 4;16:1594676. doi: 10.3389/fpls.2025.1594676 (PMC12358470; doi:10.3389/fpls.2025.1594676)
Supplement: Supplementary file 1 [file DataSheet1.docx]

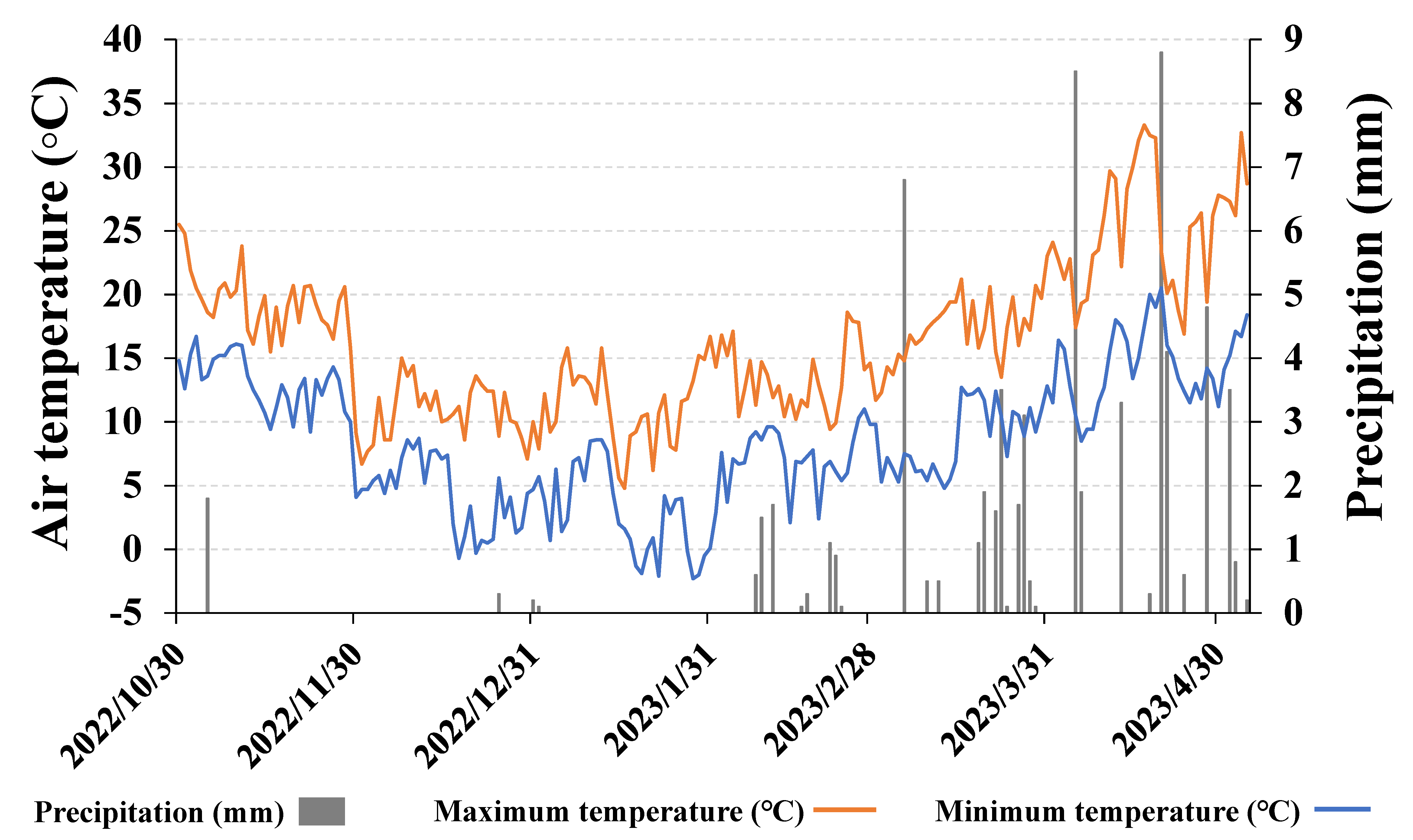


**Supplementary Figure 1 Daily precipitation (mm) and air temperature (℃) during the experiment period.**


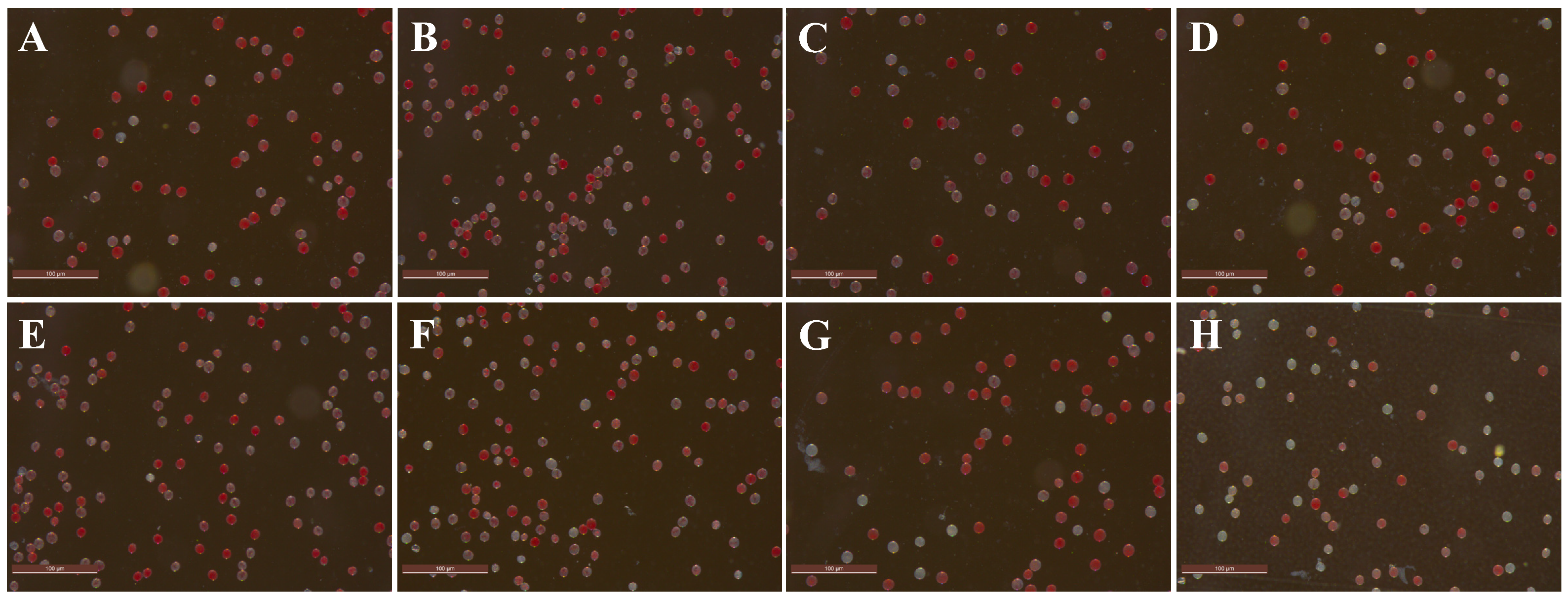


**Supplementary Figure 2 Pollen viability stained with triphenyltetrazolium chloride.** (A)~(D) Chuanmai 104, (E)~(H) Chuanmai 42.


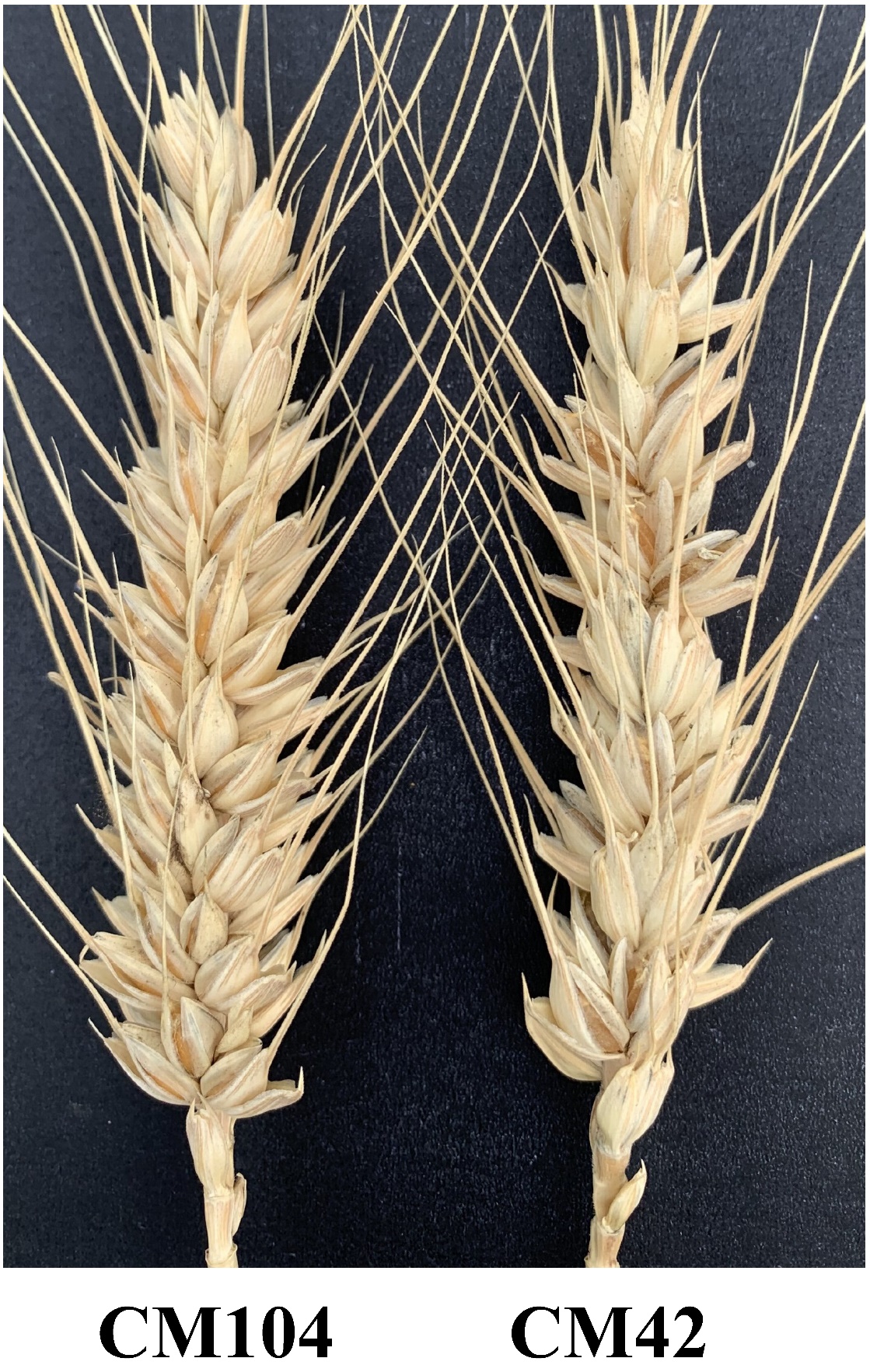


**Supplementary Figure 3 Morphology of the spike at maturity for two** **varieties after cold stress.** CM104: Chuangmai 104; CM42: Chuanmai 42**.**


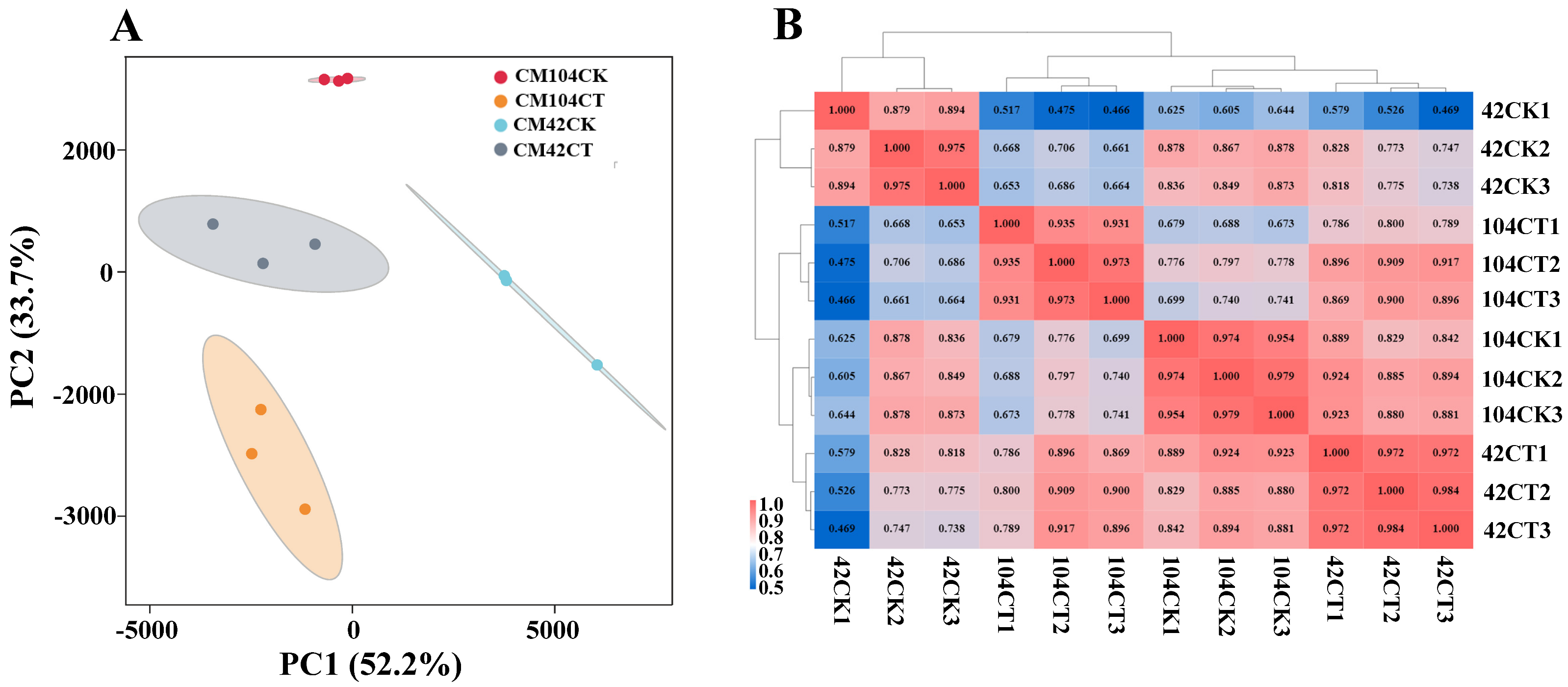


**Supplementary Figure 4 Principal component analysis (A) and clustering heatmap (B) of all samples for transcriptomic data.** Chuanmai 104_CK: 104CK1~CK3, Chuanmai 42_CK: 42CK~CK3, Chuanmai 104_CT: 104CT1~CT3, and Chuanmai 42_CT: 42CT1~CK3.


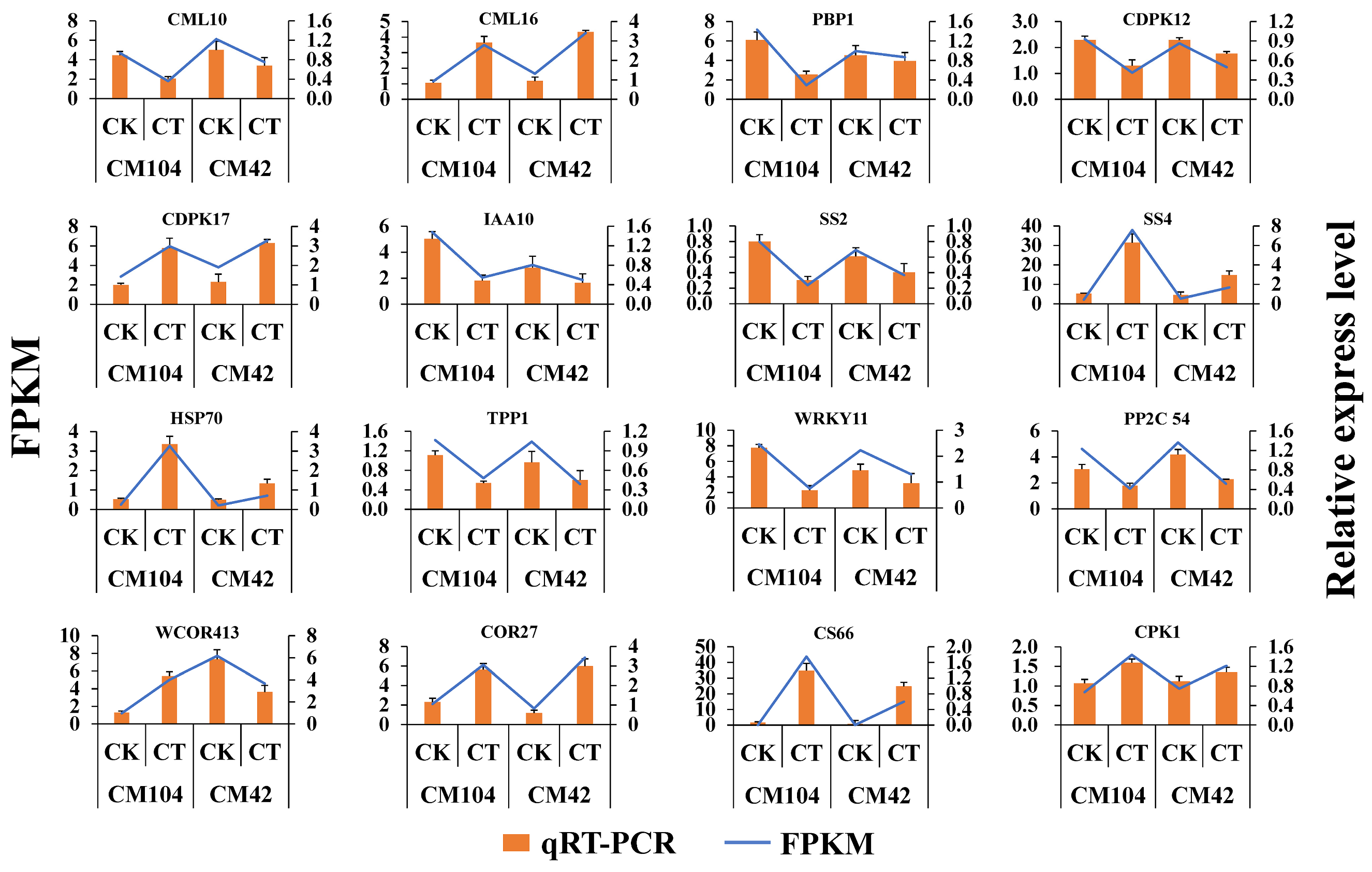


**Supplementary Figure 5 Validation of differentially expressed genes was performed using qRT-PCR.** The x-axis indicates samples in different treatments. The right y-axis indicates relative gene expression levels detected by qRT-PCR. The left y-axis indicates the FPKM value.


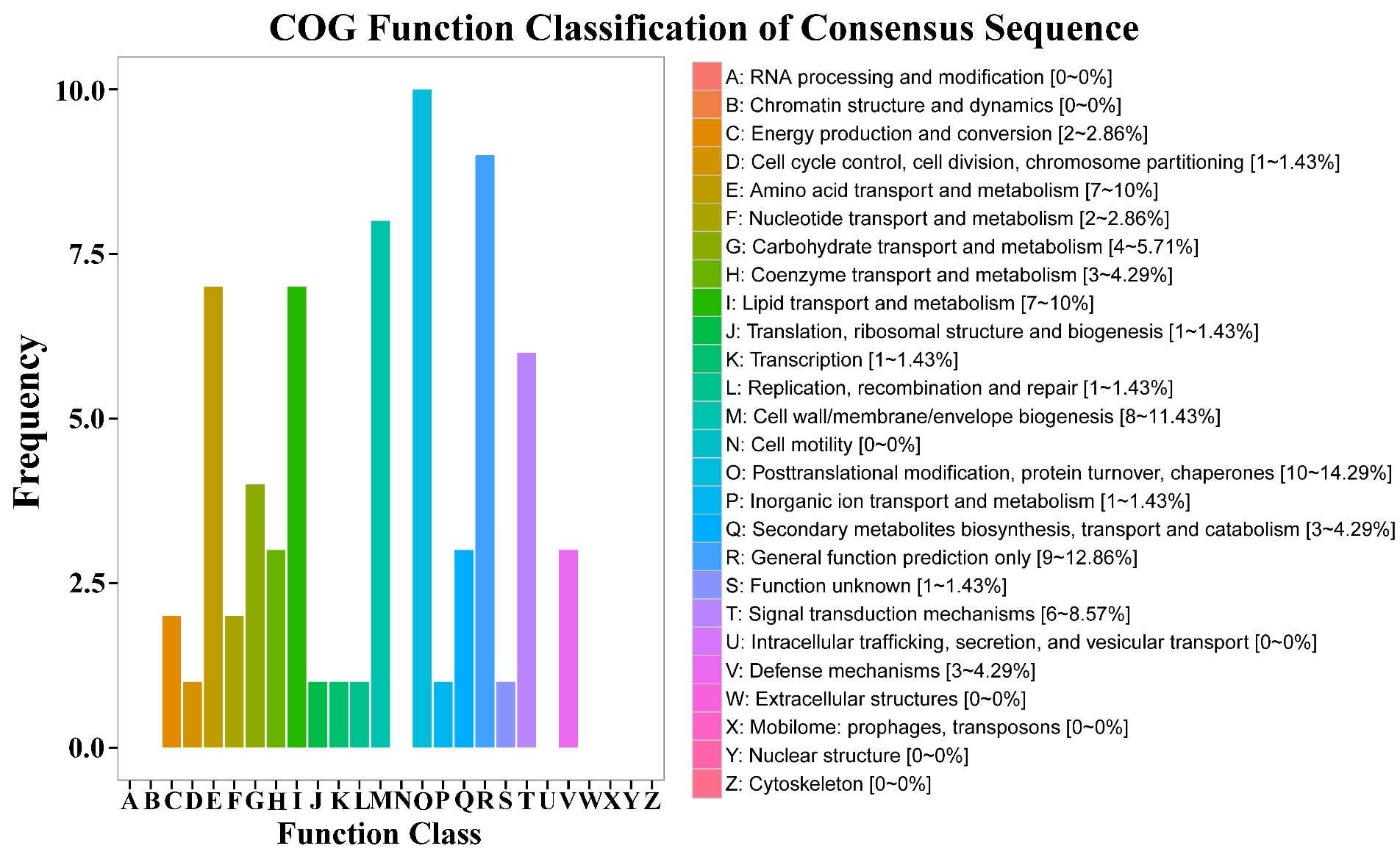


**Supplementary Figure 6 COG enrichment analysis of** **differentially expressed proteins in Chuanmai 104.**


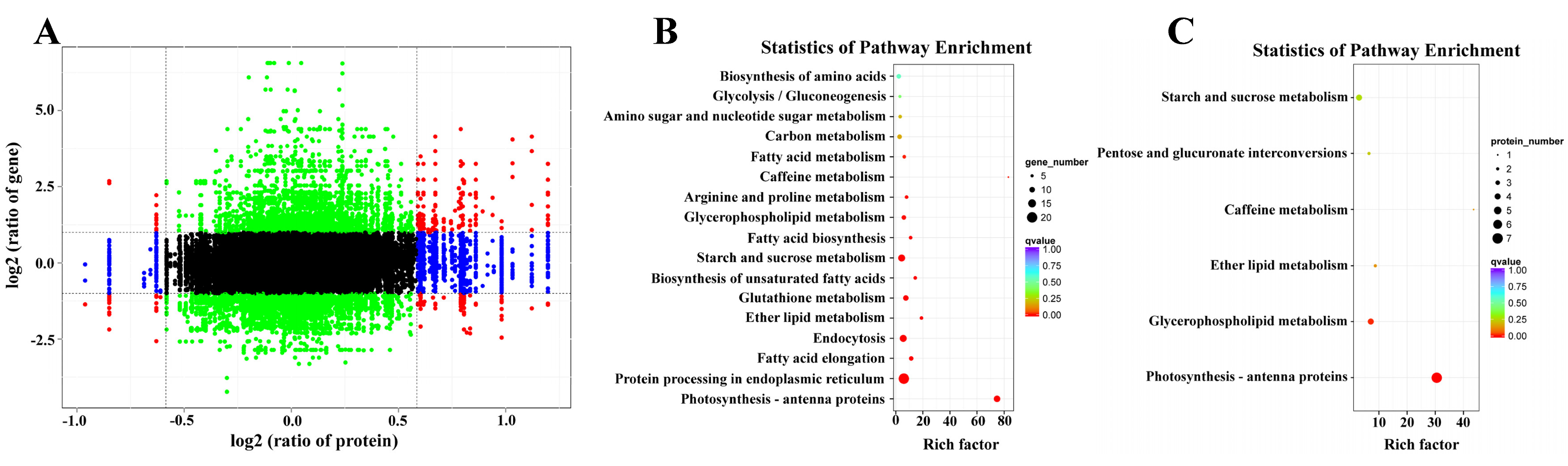


**Supplementary Figure 7 Combined analysis of the transcriptome and proteome of** **Chuanmai 104 under cold stress.** (A) Nine-quadrant diagram of differentially expressed genes (DEGs) and differentially expressed proteins (DEPs), (B) KEGG pathway enrichment analysis of DEGs, and (C) KEGG pathway enrichment analysis of DEPs.


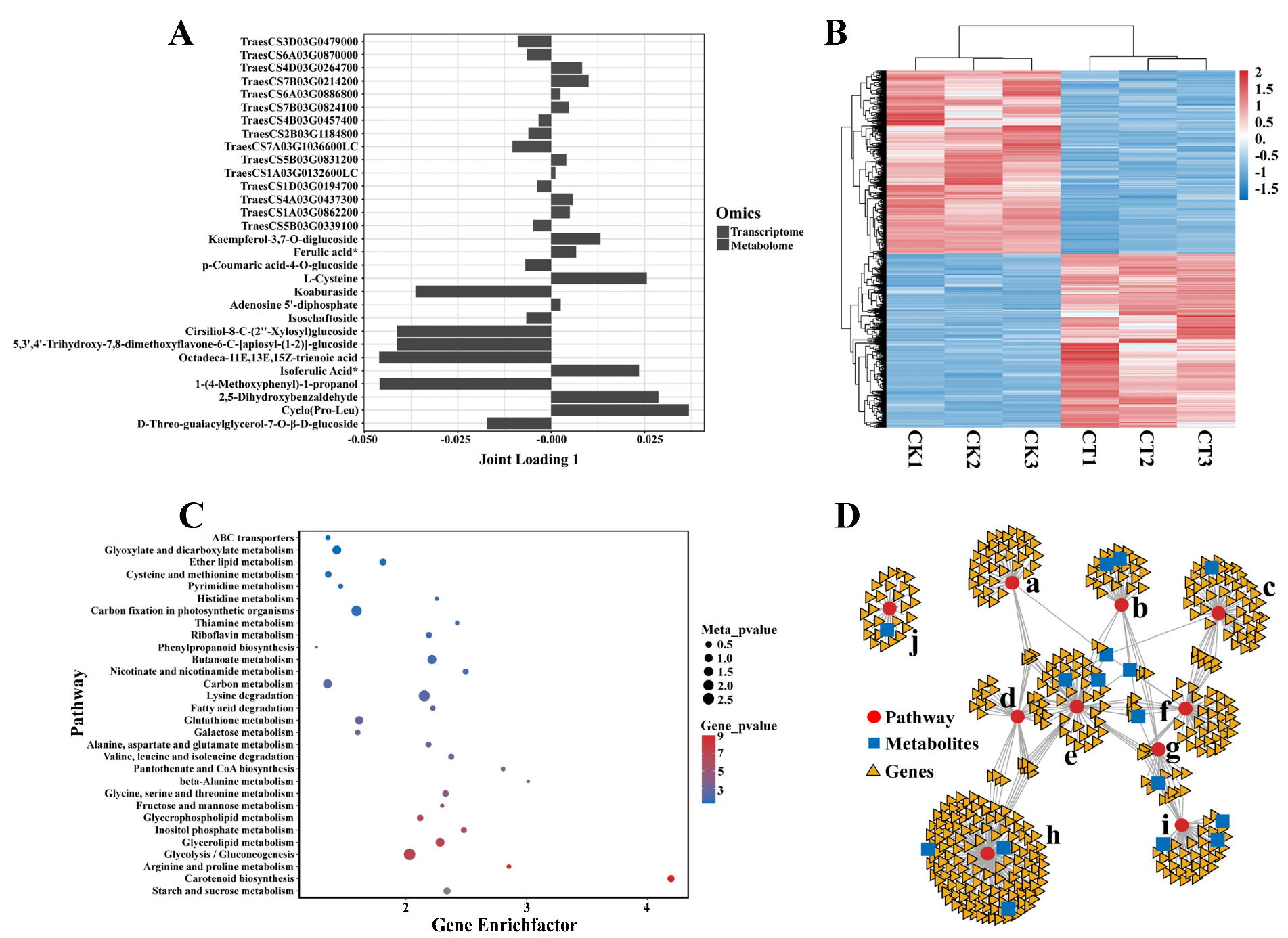


**Supplementary Figure 8 Combined analysis of transcriptome and metabolome for Chuanmai 104 under cold stress.** (A) Histogram of differentially expressed genes (DEGs)/differentially accumulated metabolites (DAMs) with high O2PLS association, (B) Clustering heatmap of DEGs/DAMs, (C) KEGG pathway enrichment analysis of DEGs/DAMs, and (D) Pathway and KGML network map of DEGs/DAMs. a: Inositol phosphate metabolism; b: Glycine, serine and threonine metabolism; c: Glycerolipid metabolism; d: Fructose and mannose metabolism; e: Glycolysis/Gluconeogenesis; f: Glycerophospholipid metabolism; g: beta-Alanine metabolism; h: Starch and sucrose metabolism; i: Arginine and proline metabolism; j: Carotenoid biosynthesis.
